# NOS inhibition reverses TLR2-induced chondrocyte dysfunction and attenuates age-related osteoarthritis

**DOI:** 10.1073/pnas.2207993120

**Published:** 2023-07-10

**Authors:** Ping Shen, Sebastian Serve, Peihua Wu, Xiaohui Liu, Yujie Dai, Nayar Durán-Hernández, Dan Thi Mai Nguyen, Michael Fuchs, Tazio Maleitzke, Marie-Jacqueline Reisener, Maria Dzamukova, Katrin Nussbaumer, Tobias M. Brunner, Yonghai Li, Vivien Holecska, Gitta A. Heinz, Frederik Heinrich, Pawel Durek, Georgia Katsoula, Clemens Gwinner, Tobias Jung, Eleftheria Zeggini, Tobias Winkler, Mir-Farzin Mashreghi, Matthias Pumberger, Carsten Perka, Max Löhning

**Affiliations:** ^a^Pitzer Laboratory of Osteoarthritis Research, German Rheumatism Research Center, a Leibniz Institute, 10117 Berlin, Germany; ^b^Experimental Immunology and Osteoarthritis Research, Department of Rheumatology and Clinical Immunology, Charité–Universitätsmedizin Berlin, Corporate Member of Freie Universität Berlin and Humboldt-Universität zu Berlin, 10117 Berlin, Germany; ^c^Stem Cell and Biotherapy Engineering Research Center of Henan Province, College of Life Sciences and Technology, Xinxiang Medical University, 453003 Xinxiang, China; ^d^Department of Orthopaedic Surgery, University of Ulm, 89081 Ulm, Germany; ^e^Center for Musculoskeletal Surgery, Charité–Universitätsmedizin Berlin, Corporate member of Freie Universität Berlin and Humboldt-Universität zu Berlin, 10117 Berlin, Germany; ^f^Julius Wolff Institute, Berlin Institute of Health at Charité–Universitätsmedizin Berlin, 13353 Berlin, Germany; ^g^Berlin Institute of Health Charité Clinician Scientist Program, BIH Biomedical Innovation Academy, Berlin Institute of Health at Charité–Universitätsmedizin, 10178 Berlin, Germany; ^h^Systems Rheumatology and Therapeutic Gene Regulation, German Rheumatism Research Center, a Leibniz Institute, 10117 Berlin, Germany; ^i^Technical University of Munich School of Medicine, Technical University of Munich, Graduate School of Experimental Medicine, 81675 Munich, Germany; ^j^Institute of Translational Genomics, Helmholtz Zentrum München – German Research Center for Environmental Health, 85764 Neuherberg, Germany; ^k^Technical University of Munich School of Medicine, Technical University of Munich and Klinikum Rechts der Isar, 81675 Munich, Germany; ^l^Berlin Institute of Health Center for Regenerative Therapies, Berlin Institute of Health at Charité ‒ Universitätsmedizin Berlin, 13353 Berlin, Germany

**Keywords:** osteoarthritis, chondrocytes, Toll-like receptors, nitric oxide synthase, cartilage-anabolic and catabolic activities

## Abstract

Osteoarthritis (OA) is a joint disease featuring cartilage breakdown and chronic pain. Although age and joint trauma are prominently associated with OA occurrence, the trigger and signaling pathways propagating their pathogenic aspects are ill defined. Following long-term catabolic activity and traumatic cartilage breakdown, debris accumulates and can trigger Toll-like receptors (TLRs). Here we show that TLR2 stimulation suppressed the expression of matrix proteins and induced an inflammatory phenotype in human chondrocytes. Further, TLR2 stimulation impaired chondrocyte mitochondrial function, resulting in severely reduced adenosine triphosphate (ATP) production. RNA-sequencing analysis revealed that TLR2 stimulation upregulated nitric oxide synthase 2 (*NOS2*) expression and downregulated mitochondria function-associated genes. NOS inhibition partially restored the expression of these genes, and rescued mitochondrial function and ATP production. Correspondingly, *Nos2*^−/−^ mice were protected from age-related OA development. Taken together, the TLR2–NOS axis promotes human chondrocyte dysfunction and murine OA development, and targeted interventions may provide therapeutic and preventive approaches in OA.

## Significance

Osteoarthritis (OA) is a chronic joint disease, in which the mechanistic trigger and facilitator remain elusive. We hypothesized that Toll-like receptors (TLRs) of chondrocytes could be activated by debris accumulating in the joint to promote OA pathology. We found that human chondrocytes express various TLRs ex vivo. In hypoxic 3D cultures, we compared side-by-side how human chondrocytes react to stimulation of TLR1 to TLR9. TLR2-mediated stimulation most drastically suppressed chondrocyte spheroid growth by increasing cartilage-catabolic and inflammatory activities and decreasing cartilage-anabolic and metabolic activities in chondrocytes. NOS inhibition partially suppressed the increased catabolism and inflammation, and rescued mitochondrial function. *Nos2* deletion protected mice from age-related OA. Thus, TLR2 signaling acts as a putative driver of OA-related chondrocyte dysfunction through NOS activity.

Osteoarthritis (OA) is a chronic degenerative joint disease hallmarked by articular cartilage breakdown, which affects more than 300 million people worldwide (1). Yet, the mechanisms that trigger and facilitate the development of OA remain elusive, impeding the development of preventive and curative treatments. Inflammatory cytokines such as interleukin 1β (IL-1β), IL-6, and tumor necrosis factor α (TNFα) are considered putative disease inducers and are frequently used as stimulators in OA models (2–4). However, interventions targeting these cytokines remained ineffective in treating OA to date (5–8). Chondrocyte dysfunction as well as cartilage breakdown are major features of OA and cause OA-related symptoms in aged and traumatized joints (9). The resulting intracapsular debris is likely a key driver of cartilage-intrinsic OA pathogenesis. Indeed, cartilage matrix degradation generates peptides including 32-mer (10) and 29 kDa fibronectin fragments (11) that act as damage-associated molecular patterns (DAMPs). Via TLR2 they promote the production of matrix metalloproteinase 3 (MMP3) and IL-6 in chondrocytes (10, 11). In addition to an imbalance in cartilage anabolism (cartilage matrix synthesis) and catabolism (cartilage matrix degradation) and enhanced inflammation, impaired mitochondrial function of chondrocytes is also a key aspect of OA, which is in part considered a metabolic disorder (12–14). However, whether TLR signaling regulates the mitochondrial respiration capacity of chondrocytes has not yet been investigated. Here we performed a comprehensive study on TLR expression in human chondrocytes ex vivo, and their responses to TLR stimulation regarding extracellular matrix homeostasis, inflammation, and energy metabolism. We demonstrate that TLR2 signaling acts as a putative driver of OA-related chondrocyte dysfunction through upregulation of nitric oxide synthase 2 (NOS2). Targeting the TLR-NOS2 axis could therapeutically rescue articular chondrocytes from excessive catabolism, inflammation, and energy deprivation.

## Results

### Human chondrocytes express various TLRs.

**Fig. 1.**
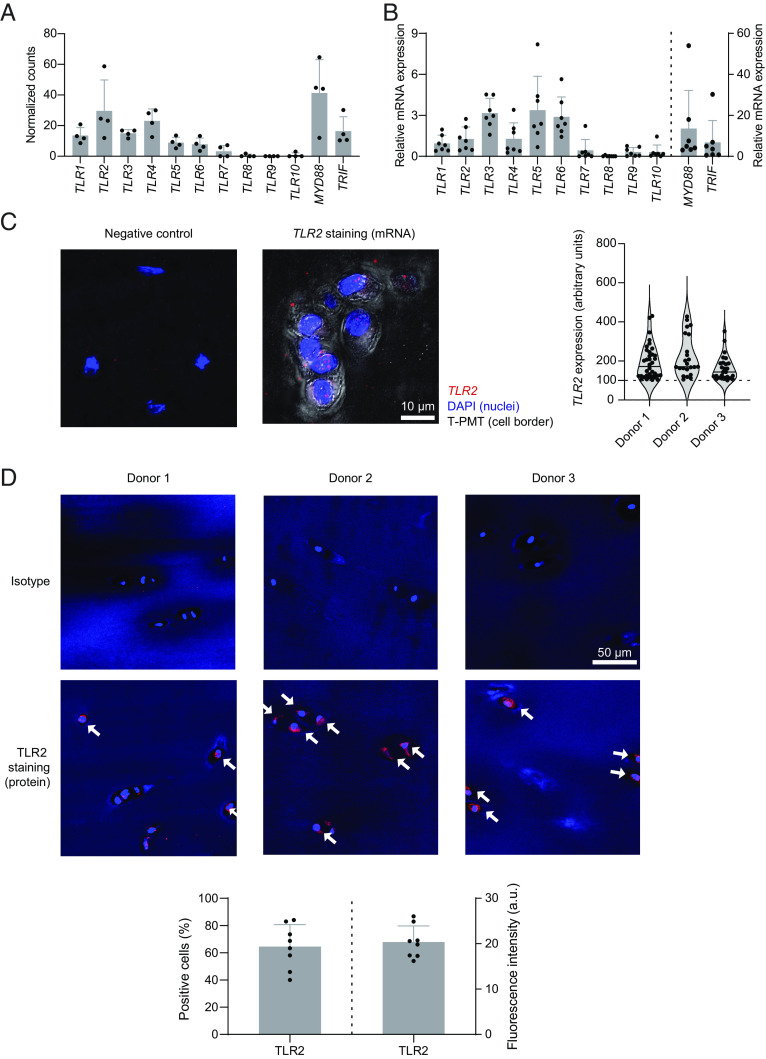
Human chondrocytes express various TLRs. (*A*) Cartilage tissue from knee joints of OA patients was dissected freshly ex vivo and immediately subjected to RNA isolation (two samples per patient). RNA-seq analysis was conducted. Normalized read counts of the duplicates were averaged. Data present averaged normalized read counts of *TLR1 to TLR10 MYD88*, and *TRIF* (n = 4, mean + SD). (*B*) TaqMan PCR was further employed to detect the expression of TLR family members, *MYD88*, and *TRIF* in fresh cartilage (n = 7, mean + SD). (*C*) Cylinders of cartilage tissue were collected freshly ex vivo and processed for RNAScope assay. Probes against the bacterial gene *DapB* were used for negative control stainings. Violin plots show *TLR2* mRNA expression in situ, using 100 arbitrary units as cutoff, in individual chondrocytes of three patients (mean ± SD). (*D*) Cartilage cylinders were collected immediately after surgery and processed for immunofluorescence analysis of TLR2 protein. The upper panel shows representative pictures of TLR2 and the corresponding isotype control staining from three donors, and the lower panel indicates the percentage of TLR2-positive cells among all the cells in the analyzed fields and the fluorescence intensity (arbitrary units) of the TLR2-positive cells (n = 8, mean + SD).

TLR stimulation has been shown to induce catabolic reactions of human chondrocytes (15–18), whereas knockout mice of TLR family genes yielded ambiguous results in experimental OA models (19, 20). To date very few studies have investigated the expression of only select TLR family members in human chondrocytes by using a histological approach (21–23). However, the interpretation of the data has remained difficult, and there is a lack of completeness. To gain a global comprehension of the expression of TLR molecules in human chondrocytes, we used a systematic approach to assess the mRNA expression of TLR family members and their signaling adaptors MyD88 and TRIF in human OA cartilage ex vivo. We isolated RNA directly from fresh cartilage tissue obtained from OA patients undergoing knee arthroplasty and performed RNA-sequencing (RNA-seq) analysis. We detected the expression of *TLR1, 2, 3, 4, 5, 6* and to a lower degree of *TLR7, 8, 9*, and *10* as well as expression of the TLR signaling adaptor molecules *MYD88* and *TRIF* (Fig. 1*A*). This observation is supported by a publicly available RNA-seq data set of knee chondrocytes from healthy and OA-diseased donors (GSE114007) (24), in which we found comparable expression of various TLR genes already in the healthy state as much as in the OA samples, indicating that chondrocytes express TLR genes prior to OA onset (*SI Appendix*, Fig. S1*A*). We further confirmed the expression of the TLR-encoding genes by TaqMan quantitative PCR (qPCR) (Fig. 1*B*). Moreover, by flow-cytometric analysis of freshly isolated human chondrocytes, we detected the expression of TLR1, 2, 4, 5, 6, and 9 at protein level (*SI Appendix*, Fig. S1 *B* and *C*). In addition, we confirmed *TLR2* mRNA expression (Fig. 1*C*) and TLR2 protein expression (Fig. 1*D*) in situ in chondrocytes of freshly obtained cartilage tissue using RNAScope hybridization and immunofluorescence analysis, respectively. Taken together, primary human chondrocytes express the genes and produce the proteins of many TLR family members ex vivo and thus are likely to respond to TLR stimulation.

### Chondrocytes Respond Differently to Distinct TLR Stimulation.

**Fig. 2.**
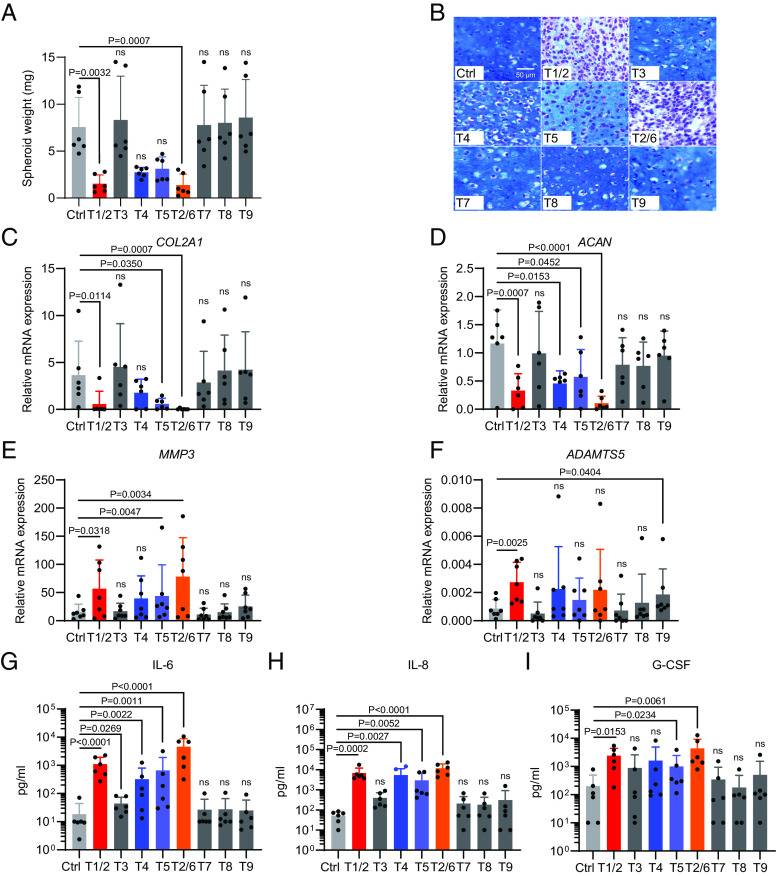
Chondrocytes respond differently to distinct TLR stimulation. Chondrocyte spheroids were generated from knee cartilage of OA patients and stimulated with agonists of TLR1/2, 3, 4, 5, 2/6, 7, 8, and 9 for 28 d. (*A*) The weight of three spheroids per condition was averaged. Combined data of independent experiments with chondrocytes derived from six OA patients are shown (n = 6, mean + SD). (*B*) Spheroids were formalin-fixed, paraffin-embedded, sectioned, and stained with Alcian blue and Nuclear Fast red. Representative images of each condition from the same patient are shown. (*C*–*F*) Spheroids were lysed for mRNA isolation. The expression of anabolic factors *COL2A1* and *ACAN* (n = 6), and catabolic factors *MMP3* and *ADAMTS5* (n = 7) was assessed by qPCR (mean + SD). (*G*–*I*) Supernatants were harvested on day 28 and analyzed using Bio-Plex assays to determine the concentrations of IL-6, IL-8, and G-CSF (n = 6, mean + SD). For statistical analysis, Friedman test was performed comparing each TLR-stimulated condition to the respective Ctrl. *P* values > 0.05 are considered as nonsignificant (ns).

**Fig. 3.**
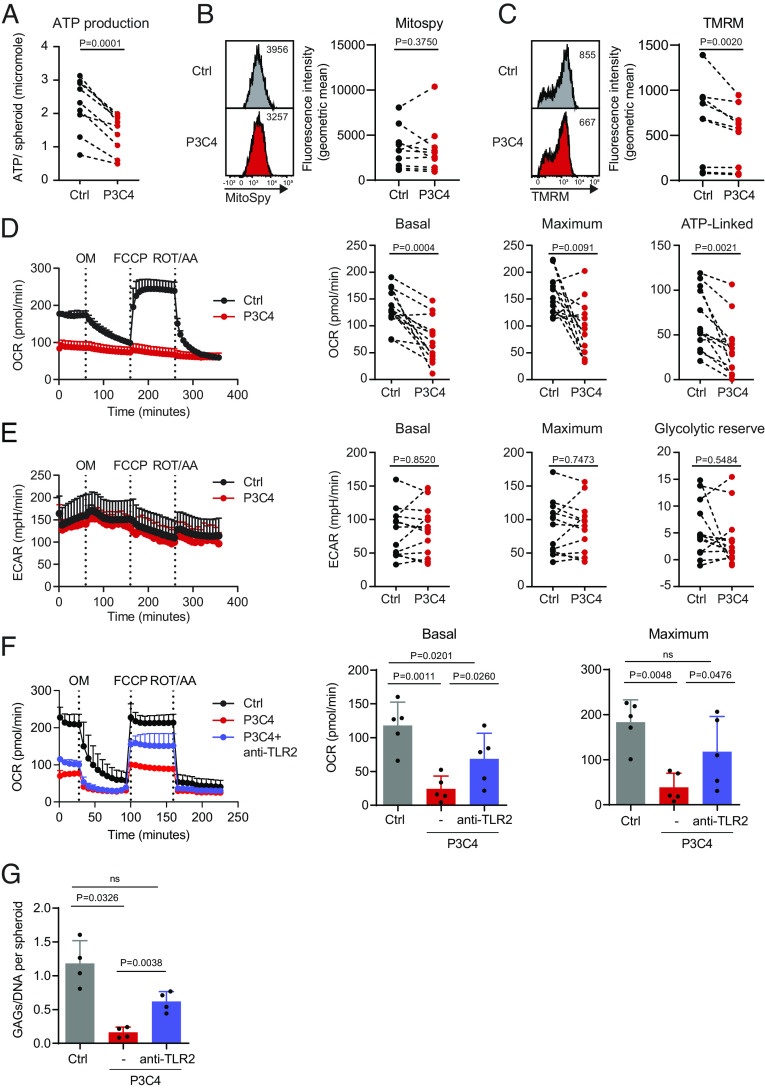
TLR1/2 stimulation impairs mitochondrial respiration of human chondrocytes, and anti-TLR2 pretreatment prevents the impairment of mitochondrial respiration and the loss of matrix components. Chondrocyte spheroids were stimulated with TLR1/2 agonist P3C4 for 3.5 d. (*A*) Spheroids were grinded and lysed individually to release ATP, which was immediately quantified and calculated as number of ATP molecules per spheroid (n = 9). (*B* and *C*) Spheroids were dissociated with collagenase II to generate single chondrocytes, which were subsequently stained with MitoSpy and TMRM. Graphs show representative stainings and fluorescence intensity of MitoSpy (*B*) and TMRM (*C*) (n = 10). (*D* and *E*) Chondrocyte spheroids were transferred individually into Seahorse XFe96 Spheroid Microplate wells and analyzed for their OXPHOS (*D*) and glycolytic activity (*E*) using Mito Stress Test Kits (n = 13). Data were compared using paired two-tailed *t* test. For (*B* and *C*), Wilcoxon matched-pairs signed-rank test was applied. (*F* and *G*) Chondrocyte spheroids were generated and preincubated with a monoclonal blocking TLR2 antibody for 3 h, prior to the addition of P3C4. After 4 d of culture, spheroids were collected for Seahorse assays (*F*, n = 5, mean + SD). Some spheroids were kept in culture until day 28. Glycosaminoglycans (GAGs) and DNA content were quantified, and the ratios of GAGs to DNA are plotted (*G*, n = 4, mean + SD). Data were analyzed with one-way ANOVA (ns, *P* > 0.05).

To evaluate the effects of TLR stimulation on primary human OA chondrocytes, we first set up a three-dimensional spheroid culture system to maintain optimal chondrogenesis (25, 26). In addition, we maintained the oxygen level at 4% to achieve a physioxia growth environment (27). To these cultures, we added agonists for TLR1/2, 3, 4, 5, 2/6, 7, 8, or 9 (for TLR10 a defined agonist is not yet available; *SI Appendix*, Fig. S2*A*). After 18 h, the expression of *NFKB,* a known target of most TLR signaling, was upregulated as assessed by qPCR (*SI Appendix*, Fig. S2*B*), confirming that human chondrocytes are responsive to TLR agonists. By the end of a 28-d culture, spheroid weights were determined. TLR1/2 and 2/6 stimulation suppressed the growth of chondrocyte spheroids most strongly, with a weight reduction of about 80%. The activation of TLR4 and 5 resulted in a less pronounced suppression, while addition of TLR3, 7, 8, and 9 agonists had no impact on spheroid growth (Fig. 2*A*). Alcian blue staining revealed the lowest accumulation of extracellular matrix (ECM) in TLR1/2- and 2/6-stimulated spheroids. The difference was less pronounced in TLR4- and 5-stimulated samples (Fig. 2*B* and *SI Appendix*, Fig. S2*C*). The reduction of ECM production elicited by TLR1/2 and TLR2/6 stimulation was further confirmed by reduced glycosaminoglycans (GAGs)/DNA ratios of each spheroid after a 28-d culture (*SI Appendix*, Fig. S2*D*). This pattern in the reduction of spheroid growth was reflected by the diminished expression of transcripts encoding the matrix proteins collagen 2A1 (*COL2A1*) and aggrecan (*ACAN*), as well as enhanced expression of the cartilage-degrading enzymes *MMP3* and a disintegrin and metalloproteinase with thrombospondin motifs 5 (*ADAMTS5*) (Fig. 2 *C*–*F*). Beyond the previously reported IL-6 induction by TLR2 (16, 17), we found a broader effect of TLR stimulation on the inflammatory phenotype of chondrocytes: TLR1/2 and TLR2/6 stimulation induced secretion of inflammatory cytokines including macrophage inflammatory protein-1α (MIP-1α/CCL3), MIP-1ß (CCL4), TNFα, interferon-γ (IFNγ), and triggered high-level production of IL-6, IL-8, and granulocyte colony-stimulating factor (G-CSF) (Fig. 2 *G*−*I* and *SI Appendix*, Fig. S2*E*). Of note, in many aspects, TLR1/2 stimulation and TLR2/6 stimulation elicited very similar effects: 1) A similar inhibition on chondrocyte spheroid growth (Fig. 2 *A* and *B* and *SI Appendix*, Fig. S2 *C* and *D*); 2) a similar suppression of expression of the cartilage-anabolic factors *COL2A1* and *ACAN* and a similar enhancement of the catabolic factors *MMP3* and *ADAMTS5* (Fig. 2 *C*-*F*); 3) a similar induction of the inflammatory cytokines IL-6, IL-8, G-CSF and other cytokine secretion (Fig. 2 *G*−*I* and *SI Appendix*, Fig. S2*E*). Thus, TLR2 signaling, which is elicited downstream of both TLR complexes with TLR2 participation, i.e., TLR1/2 and TLR2/6, strongly impairs ECM generation while simultaneously inducing a proinflammatory phenotype in human chondrocytes.

Considering these observations, we further investigated the function of TLR1/2 signaling in suppressing chondrocyte spheroid growth, starting with a kinetic analysis of a 4-wk culture, in which spheroids were sampled every 3.5 d. Stimulation with the TLR1/2 agonist Pam3CSK4 (P3C4) suppressed the growth of spheroids from early on, which was associated with decreased *COL2A1* and increased *MMP3* expression (*SI Appendix*, Fig. S3 *A*–*C*). P3C4 also tended to increase the secretion of the inflammatory cytokines IL-6, IL-8, and G-CSF throughout the stimulation period (*SI Appendix*, Fig. S3 *D*–*F*).

### TLR1/2 Stimulation Impairs Mitochondrial Respiration of Human Chondrocytes.

Since OA chondrocytes have been reported to feature reduced ATP production (12), we assessed whether TLR1/2 stimulation was sufficient to induce energy deficiency in human chondrocytes. Four days after stimulation with P3C4, spheroid cellular ATP production was significantly reduced compared to control spheroids (Fig. 3*A*). Comparable MitoSpy staining intensities between control and P3C4-stimulated spheroids indicated a similar total mitochondrial mass per cell (Fig. 3*B*). However, a significant reduction of the mitochondrial membrane potential was detected by TMRM staining (Fig. 3*C*). Mitochondrial membrane potential is a key indicator of mitochondrial activity because it reflects the process of electron transport and oxidative phosphorylation (OXPHOS), the driving forces behind mitochondrial ATP production (28). We therefore conducted a Seahorse Spheroid Mito Stress Test on day 4 of culture, when the spheroid weight and chondrocyte cell number, viability, and expansion rates were not yet affected by P3C4 stimulation (*SI Appendix*, Fig. S3 *A* and *G*–*I*). Similar matrix densities (*SI Appendix*, Fig. S3*J*) likely allowed comparable spheroid infiltration of oligomycin, FCCP, and rotenone/actinomycin A, which are used to quantify OXPHOS activity and glycolytic efficiency by assessing the oxygen consumption rate (OCR) and extracellular acidification rate (ECAR). P3C4 stimulation strongly diminished OXPHOS activity-as shown by the reduction of both basal and maximum OCR – and ATP-linked oxygen consumption (Fig. 3*D*). Basal and maximum glycolytic rates as well as glycolytic reserves were comparable, indicating that glycolysis was not affected (Fig. 3*E*). To confirm that the observed stimulatory effects of P3C4 were propagated by TLR2, we treated chondrocytes with a blocking antibody against human TLR2, prior to P3C4 stimulation. Indeed, TLR2 antibody pretreatment largely prevented the P3C4-mediated impairment of mitochondrial respiration (Fig. 3*F*), as well as the loss of spheroid matrix components (Fig. 3*G*). To investigate whether TLR1/2 stimulation also has an impact on mature chondrocyte spheroids, we first cultured chondrocyte spheroids for 4 wk to establish maturity, then added P3C4, and performed metabolic analyses 4 d later. TLR1/2 stimulation impaired the OXPHOS activity without affecting glycolysis (*SI Appendix*, Fig. S4*A*) and suppressed total cellular ATP production also in mature chondrocyte spheroids (*SI Appendix*, Fig. S4*B*). Furthermore, *COL2A1* and *ACAN* expression decreased, while *MMP3* and *ADAMTS5* expression increased (*SI Appendix*, Fig. S4*C*) together with IL-6, IL-8, and G-CSF (*SI Appendix*, Fig. S4 *D*–*G*). Thus, P3C4 stimulation of mature chondrocyte spheroids for 4 d is sufficient to suppress ECM anabolic gene expression, enhance transcription of catabolic genes, and induce inflammatory cytokine secretion. In addition, P3C4 stimulation selectively impairs mitochondrial respiration but not glycolysis in both mature and just 4-d–old chondrocyte spheroids.

**Fig. 4.**
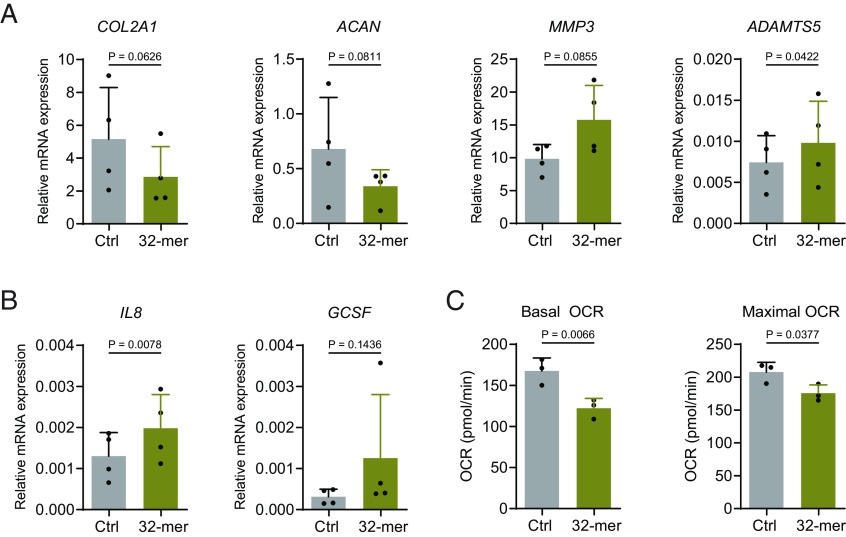
32-mer treatment tends to reduce anabolic factor expression and metabolic activity and to increase the expression of catabolic and inflammatory factors. 32-mer peptide of aggrecan was added to chondrocyte spheroid cultures. On day 4, spheroids were harvested for mRNA detection (*A* and *B*, n = 4, mean + SD) or for Seahorse assay (*C*, n = 3, mean + SD). Data were analyzed with one-tailed paired *t* test.

We have described above the detrimental effect of P3C4-mediated TLR1/2 stimulation on human chondrocytes. To investigate the OA-associated pathogenic impact of TLR1/2 signaling triggered by an endogenously occurring agonist, we stimulated chondrocyte spheroids with 32-mer, which is a peptide released from the matrix protein aggrecan after several enzymatic digestion steps and which can activate TLR2 (10). We observed that 32-mer tended to reduce the expression of *COL2A1* and *ACAN* and increase the expression of *MMP3* and *ADAMTS5* (Fig. 4*A*) and of the inflammatory cytokines *IL8* and *GCSF* (Fig. 4*B*). In addition, 32-mer significantly reduced the mitochondrial respiration capacity of chondrocytes (Fig. 4*C*). Overall, endogenous 32-mer stimulation yielded similar results as P3C4 stimulation, although the effect of 32-mer was less pronounced when compared to P3C4.

### TLR1/2-Induced Chondrocyte Dysfunction Is Not Mediated by Enhanced ROS Accumulation or Reduced Mitochondrial Respiration.

**Fig. 5.**
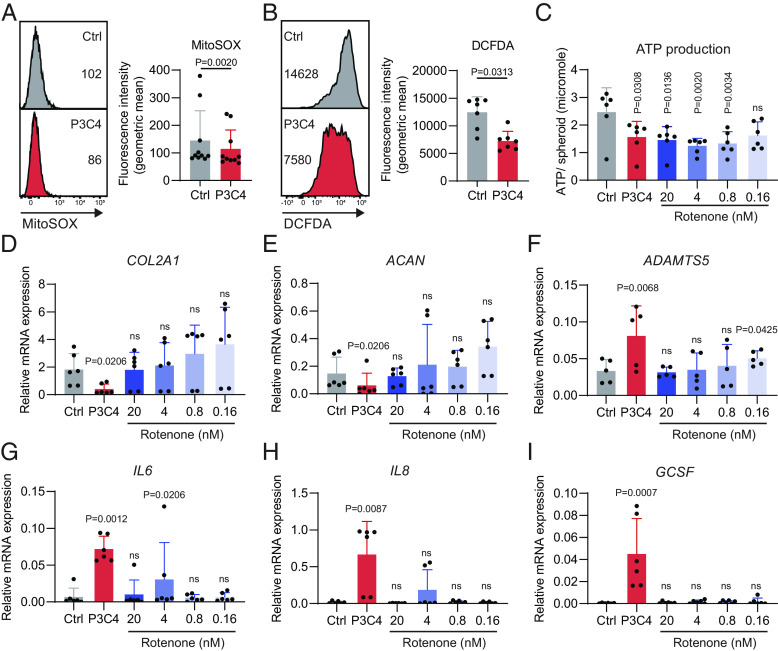
TLR1/2-induced chondrocyte dysfunction is not mediated by enhanced ROS accumulation or reduced mitochondrial respiration. Chondrocyte spheroids were stimulated with P3C4 for 3.5 d. Spheroids were then dissociated with collagenase II to generate single chondrocytes, which were stained with MitoSOX (*A*) and DCFDA (*B*), separately. *Left*, Representative FACS plots. *Right*, geometric fluorescence intensity of MitoSOX (n = 10) or DCFDA (n = 7). Data (mean + SD) were analyzed using two-tailed Wilcoxon matched-pairs test. (*C*–*I*) Chondrocyte spheroids were generated and stimulated with P3C4 for 3.5 d. Decreasing concentrations of rotenone were added to inhibit mitochondrial respiration. Spheroids were grinded and lysed to quantify ATP production (*C*). Spheroids were lysed for mRNA isolation. *COL2A1, ACAN, ADAMTS5, IL6, IL8,* and *GCSF* expression was determined by qPCR (*D*–*I*). (*C*–*I*) Data (n = 6, mean + SD) were analyzed using Friedman test to compare each TLR-stimulated condition to the respective Ctrl. *P* values > 0.05 are considered as nonsignificant (ns).

Increased mitochondrial reactive oxygen species (mROS) results in oxidation of mitochondrial proteins and thus mitochondrial dysfunction (29). Mitochondria-targeted antioxidants attenuated cholesterol-induced OA (30). In addition, it has been reported that mROS promote TLR-induced inflammatory cytokine production (31–34). To understand whether the impaired mitochondrial function and the enhanced inflammatory phenotype induced by TLR1/2 stimulation resulted from over-accumulation of mROS, we measured mROS using MitoSOX staining. In contrast to our hypothesis, this analysis showed mildly but significantly reduced mROS levels in P3C4-stimulated chondrocytes (Fig. 5*A*). Next, we determined total cellular ROS production via DCFDA staining and found a reduced total cellular ROS production upon TLR1/2 stimulation (Fig. 5*B*). Thus, neither mROS nor total cellular ROS appear to be the cause of TLR1/2-induced mitochondrial dysfunction and inflammatory cytokine secretion.

Energy deficiency has been reported to metabolically hinder type II collagen synthesis during murine growth plate development (35). To determine whether the reduced mitochondrial ATP production caused the perturbation of ECM homeostasis and the inflammatory cytokine production induced by P3C4 stimulation, we partially suppressed mitochondrial respiration by adding decreasing amounts of the complex I inhibitor rotenone. At concentrations of 0.8 to 20 nM, rotenone treatment reduced the chondrocytes’ ATP production to a similar extent as TLR1/2 stimulation without affecting cell survival (Fig. 5*C* and *SI Appendix*, Fig. S5*A*). Yet, rotenone influenced neither the expression of ECM-anabolic and -catabolic factors (Fig. 5 *D*–*F*), nor the expression and secretion of inflammatory cytokines (Fig. 5 *G*–*I* and *SI Appendix*, Fig. S5*B*). Thus, the reduction of ATP production is not sufficient to recapitulate the TLR1/2-induced cartilage-degrading and inflammatory phenotype in chondrocytes.

### TLR1/2 Stimulation Promotes Nitric Oxide Production and Reduces Metabolic Gene Expression.

**Fig. 6.**
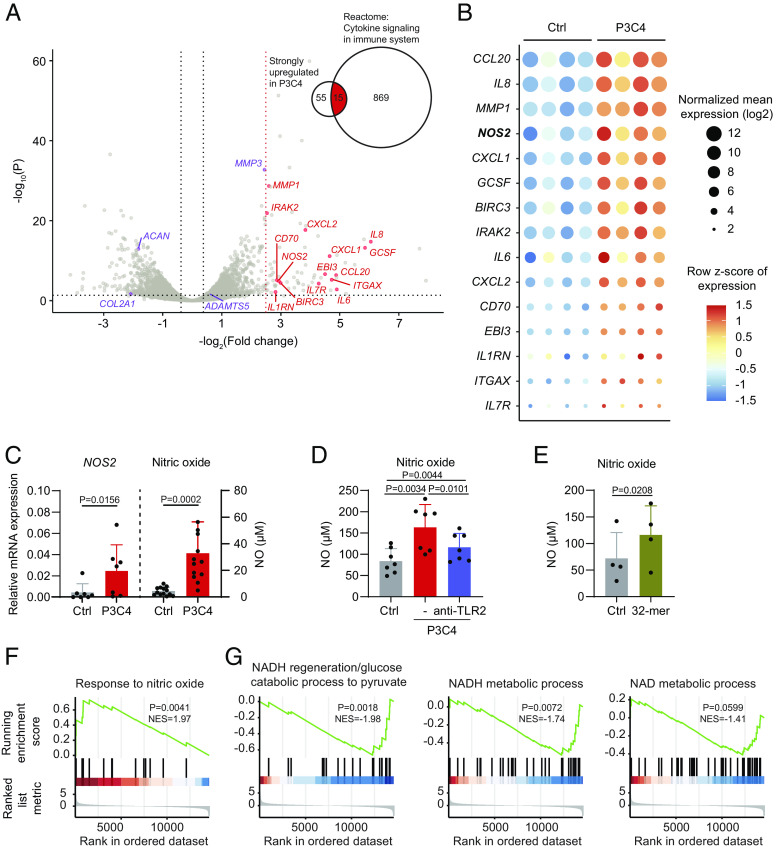
TLR1/2 stimulation promotes nitric oxide production and reduces metabolic gene expression. (*A*) RNA-seq was performed on human chondrocyte spheroids that were cultured with or without P3C4 for 3.5 d. Differentially expressed genes are plotted in a volcano plot, in which genes encoding cartilage-anabolic and catabolic factors are colored in purple and the top 15 hits of genes belonging to the gene set “cytokine signaling in immune system” are colored in red. (*B*) Heatmap displaying the expression of the top 15 hits of genes belonging to the gene set “cytokine signaling in immune system” per patient sample. (*C*) Expression of *NOS2* mRNA (n = 7) and production of nitric oxide (n = 12) were determined in chondrocyte spheroid cells and supernatants, respectively. Data (mean + SD) were analyzed using two-tailed Wilcoxon matched-pairs test. (*D*) Chondrocyte spheroids were preincubated with a blocking TLR2 antibody for three hours, prior to the addition of P3C4. After 4 d of culture, supernatants were harvested for NO quantification (n = 7, mean + SD). (*E*) 32-mer peptide was added to chondrocyte spheroids. On day 4, supernatants were collected for NO quantification (n = 4, mean + SD). Data were analyzed with one-tailed paired *t* test. (*F* and *G*) Gene set enrichment analysis was performed on four Gene Ontology gene sets based on a ranked gene list with the log2(Fold change) as underlying metric. *P* values and normalized enrichment scores (NES) are given per gene set.

To identify molecular mediator(s) responsible for the TLR1/2-induced dysfunction of chondrocytes, we sequenced RNA isolated from 3.5-d–old P3C4-stimulated and unstimulated spheroids. We found that 1,462 genes (including *MMP3* and *ADAMTS5*) were significantly upregulated and 1,419 genes (including *COL2A1* and *ACAN*) were significantly downregulated (FDR < 0.05 and fold change > 1.3) in P3C4-stimulated spheroids (Fig. 6*A*). As expected, the upregulated genes were enriched in inflammatory pathways of the immune system (*SI Appendix*, Fig. S6*A*). Importantly, *NOS2*, together with the afore-detected inflammatory cytokines *IL6*, *IL8*, and *GCSF* were among the top 15 differentially upregulated genes that are also part of the Reactome gene set “cytokine signaling in the immune system” (R-HSA-1280215) (Fig. 6 *A* and *B*). Since nitric oxide (NO) has been shown to modulate mitochondrial respiration in macrophages and chondrocytes (36–39), we quantified the upregulation of *NOS2* expression in the spheroids and the increase of NO production in the supernatants of P3C4-stimulated chondrocytes (Fig. 6*C*). This NO induction was partially prevented by preblocking TLR2 signaling via anti-TLR2 indicating that P3C4 increased NO production indeed through activating TLR1/2 (Fig. 6*D*). Moreover, the endogenous TLR2 activator 32-mer also increased the NO production of human chondrocytes (Fig. 6*E*). Taken together, TLR1/2 stimulation promotes the NO production capacity of human chondrocytes. Gene set enrichment analysis confirmed a significant positive enrichment of genes that represent a response to NO (Fig. 6*F*). Concurrent with impaired mitochondrial respiration and reduced matrix density following TLR1/2 stimulation, the downregulated genes were enriched for terms relevant to metabolic regulation, matrix organization, and translational regulation (*SI Appendix*, Fig. S6*B*). In addition, gene set enrichment analysis revealed a significant negative enrichment in gene sets related to a variety of metabolic processes (Fig. 6*G*), which provides molecular support for the observed mitochondrial dysfunction and energy deficiency induced by TLR1/2 stimulation. Interestingly, here we detected a downregulation of the NADPH oxidase 4 (*NOX4*), an important source of ROS (40), and an upregulation of the antioxidant thioredoxin reductase 1 (*TXNRD1*) (41) and the mitochondrial ROS-specific scavenger superoxide dismutase 2 (*SOD2*) (42) (*SI Appendix*, Fig. S6*C*). This may explain, at least partially, why TLR1/2 stimulation reduced ROS accumulation in chondrocytes (cf. Fig. 5 *A* and *B*).

### NOS Inhibition Restores the Expression of Mitochondrial Genes and Mitochondrial Function.

**Fig. 7.**
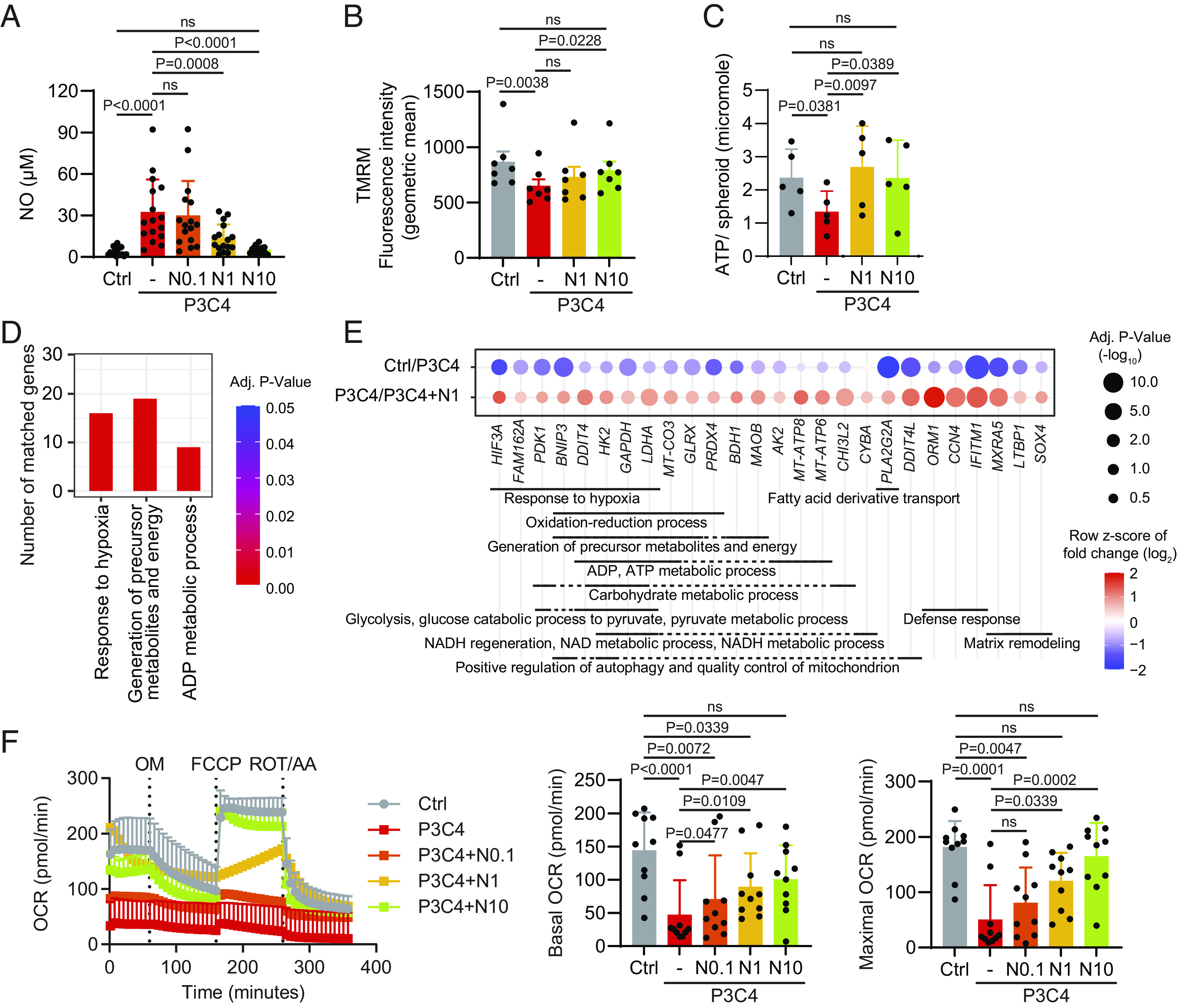
NOS inhibition restores mitochondrial gene expression and function. Chondrocyte spheroids were cultured with or without P3C4 for 3.5 d. In parallel, increasing amounts of NOS inhibitor L-NAME (N) were added to the P3C4-stimulated cultures (N0.1: 0.1 mM; N1: 1 mM; N10: 10 mM). (*A*) Nitric oxide content in the supernatants was determined by Griess reaction assay (n = 16, mean + SD). (*B*) Mitochondrial membrane potential of spheroid chondrocytes was assessed via TMRM staining (n = 7, mean + SD). (*C*) Intracellular ATP production of spheroid chondrocytes was quantified (n = 5, mean + SD). (*D*) RNA-seq analysis was performed for spheroids that were unstimulated (Ctrl) or stimulated with P3C4 or with P3C4+N1. Overrepresentation analysis on genes downregulated by P3C4 and then restored by addition of L-NAME (N1) on all “gene ontology biological processes” gene sets, of which three pathways that relate to oxygen consumption and metabolism have been selected among the gene sets with a FDR < 0.05. (*E*) The 26 mitochondroid genes that were downregulated by P3C4 and restored by addition of N1 were grouped according to their biological functions. (*F*) Spheroids were transferred individually into the Seahorse XFe96 Spheroid Microplate and analyzed for their OXPHOS activity using Mito Stress Test kits (n = 10, mean + SD). For *A*–*C* and *F*, statistical analyses were done using Friedman test. *P* values > 0.05 are considered as nonsignificant (ns).

TLR2 stimulation as well as TLR4 and 5 stimulation, increased *NOS2* expression, enhanced NO production (*SI Appendix*, Fig. S7 *A* and *B*), and impaired mitochondrial respiration in chondrocytes (*SI Appendix*, Fig. S7 *C* and *D*). This was in line with the reduced expression of cartilage-anabolic mediators and increased expression of cartilage-catabolic and inflammatory factors along with the suppressed growth of chondrocyte spheroids (cf. Fig. 2). In contrast, stimulation with TLR3, 7, 8, or 9 neither affected *NOS2* expression, NO production, nor mitochondrial function (*SI Appendix*, Fig. S7). To determine whether the increased production of NO was the cause of the impaired mitochondrial function and the enhanced inflammatory phenotype induced by TLR1/2 stimulation, we added the NOS inhibitor L-NAME to P3C4-stimulated chondrocyte spheroids. We observed a dose-dependent suppression of NO production, and NO levels comparable to controls were achieved at 10 µM L-NAME (Fig. 7*A*). Likewise, blockade of NO production profoundly rescued mitochondrial membrane potential (Fig. 7*B*), and restored the ATP production (Fig. 7*C*). Moreover, the addition of L-NAME restored the expression of 250 genes that were altered by P3C4 stimulation (*SI Appendix*, Fig. S8*A*). Among the restored genes, 42 genes functionally belong to the mitochondrial gene set (*SI Appendix*, Fig. S8*B*), suggesting that NO plays an important role in TLR1/2-mediated mitochondrial dysfunction. Functional enrichment analysis (GSEA) of the 26 mitochondroid genes that were downregulated by P3C4 and then restored by L-NAME addition revealed that these genes were involved in hypoxia response, the generation of precursor metabolites and energy, and ADP metabolic processes (Fig. 7 *D* and *E* and *SI Appendix*, Fig. S8*B*). Blockade of NO production dose-dependently restored OXPHOS activity, as both basal and maximal OCR values gradually recovered (Fig. 7*F*). Addition of the NOS inhibitor did not affect glycolysis or mROS accumulation (*SI Appendix*, Fig. S8 *C* and *D*).

### NOS Inhibition Attenuates Inflammation and Age-Related OA.

**Fig. 8.**
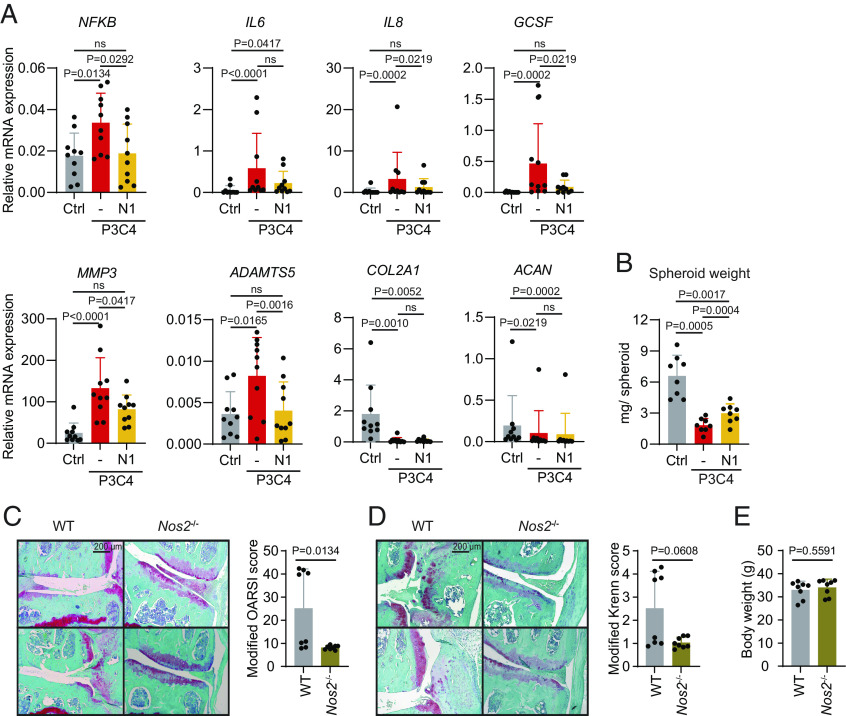
NOS inhibition attenuates inflammation and prevents age-related OA. (*A*) Chondrocyte spheroids were cultured with or without P3C4 for 3.5 d. In parallel, 1 mM NOS inhibitor L-NAME (N) were added to the P3C4-stimulated cultures. Unstimulated spheroids (Ctrl), together with those stimulated with P3C4 or P3C4+N1 were processed for mRNA isolation. Expression of *NFKB, IL6, IL8, GCSF, MMP3, ADAMTS5, COL2A1*, and *ACAN* was determined by qPCR (n = 10, mean + SD). (*B*) Spheroids that were unstimulated (Ctrl) or stimulated with P3C4 or with P3C4+N1 were cultured for 28 d. The weight of each spheroid was determined (n = 8, mean + SD). For panels *A* and *B*, statistical analyses were done using Friedman test. *P* values > 0.05 are considered as nonsignificant (ns). (*C*–*E*) C57BL/6 (WT) and *Nos2*^−/−^ mice were kept until 2 y of age. Knee OA severity and body weight were determined. Representative images of Safranin-O/Fast green staining of knee joints and modified OARSI scores (*C*), representative images of Safranin-O/Fast green staining of knee joints and modified Krenn scores to assess synovitis (*D*), as well as body weight (*E*) of WT and *Nos2*^−/−^ mice (n = 8, mean + SD) are shown. Modified OARSI and Krenn scores were compared using Mann–Whitney *U* test, and weight comparison was performed using unpaired two-tailed *t* test.

Furthermore, NOS inhibition significantly reduced the expression of TLR1/2-induced inflammatory factors (*NFKB*, *IL6*, *IL8*, and *GCSF*), and ECM-catabolic factors (*MMP3* and *ADAMTS5*) (Fig. 8*A*). However, it failed to rescue the expression of *COL2A1* and *ACAN* (Fig. 8*A*). Nevertheless, L-NAME restored the growth of chondrocyte spheroids to a significant extent (Fig. 8*B* and *SI Appendix*, Fig. S9). Importantly, NOS depletion also showed cartilage-protective effects *in vivo*. When applying histopathological scorings on 2-y–old wildtype (WT) and *Nos2*^−/−^ mice, we observed that *Nos2*^−/−^ mice exhibited less signs of OA compared to WT animals, with reduced cartilage erosion (Fig. 8*C*) and synovitis severity (Fig. 8*D*). In line with previous reports on varying incidences of naturally occurring OA in mice (43, 44), about half of the WT mice developed a fully osteoarthritic phenotype, which did not occur in *Nos2*^−/−^ mice. Thus, 2-y–old *Nos2*^−/−^ mice were significantly less affected by age-related OA compared to WT mice despite similar body weight (Fig. 8*E*), indicating a role for NOS2 in age-related OA development.

## Discussion

In summary, our data show that human primary chondrocytes express various TLRs, and upon stimulation, TLR1/2 and 2/6 most strongly suppressed the growth of chondrocyte spheroids, while TLR4 and 5 caused a less pronounced suppression, and TLR3, 7, 8, and 9 had little impact. In particular, activation of the TLR2 signaling cascades exerted strong detrimental effects on spheroid growth by inhibiting ECM synthesis and promoting ECM degradation. Further, TLR2 signaling triggered the secretion of inflammatory cytokines and impaired mitochondrial respiration (*SI Appendix*, Fig. S10, Graphical abstract). Moreover, we identified NO as an important molecular mediator of the TLR2-induced chondrocyte dysfunction: NO blockade restored mitochondrial respiration, reduced the expression of inflammatory factors and cartilage-degrading enzymes, and partially rescued spheroid growth in TLR2-stimulated chondrocytes. In accordance, *Nos2*-deficient mice were largely protected from age-related OA development.

With regard to the contribution of TLR signaling to OA development, investigations in mice using OA models induced by pure surgical approach (partial removal of the medial meniscus) have shown that deficiency of *Tlr1*, *Tlr2*, *Tlr4*, *Tlr6*, or *Myd88* had no impact on the severity of experimental OA (19). Notably, however, when the surgical OA model was exacerbated by a low-grade inflammation induced by high-fat diet, the knockout of *Tlr2* as well as of the TLR cofactors *Lbp* or *Cd14*, but not of *Tlr4*, ameliorated the OA severity (45). This indicates a pathogenic role of *Tlr2* in low-grade inflammation-exacerbated OA in mice. The low-grade inflammation associated with OA is often attributed to innate immune cells, including synovial macrophages and neutrophils, in part with support from the complement system (46–49). Indeed, high-fat diet increased the level of inflammation by inducing the infiltration of macrophages to the synovial membrane (45). In our study, we demonstrate that human chondrocytes themselves may directly contribute to the low-grade innate immune response via TLRs. Upon TLR activation, chondrocytes secreted a broad range of inflammatory mediators. Among those, the cytokines TNFα, IFNγ, IL-6, IL-8, and G-CSF are known to either directly activate chondrocytes promoting cartilage breakdown or attract macrophages and neutrophils into the synovial membrane causing synovitis. Synovitis in turn drives further cartilage breakdown (50), thereby generating more TLR agonists. These interactive inflammatory cascades mediated by chondrocytes in cartilage together with macrophages and neutrophils in the synovial membrane likely form a positive feedback system, which may promote OA development.

OA chondrocytes feature reduced oxygen consumption and lower ATP production (12), highlighting metabolic changes as an important aspect of the underlying pathophysiology. In our study, TLR1/2 stimulation impaired mitochondrial OXPHOS activity and reduced the chondrocytes’ ATP content by approximately 40%. In macrophages and cancer cells, reduced OXPHOS is compensated by enhanced glycolysis (36, 51). However, in chondrocytes TLR1/2 stimulation did not affect glycolysis efficiency, even though it reduced the expression of genes encoding key enzymes involved in glycolysis, such as hexokinase 2 (*HK2*), glyceraldehyde 3-phosphate dehydrogenase (*GAPDH*), and lactate dehydrogenase A (*LDHA*). This may be explained by the largely inert state of chondrocytes in adult human cartilage maintaining a low turnover of the matrix (52, 53), whereas macrophages and cancer cells require more energy to fuel migration and proliferation. Importantly, the ~40% decrease in intracellular ATP appears not to affect the viability of chondrocytes (cf. *SI Appendix*, Fig. S3*H*). Recent studies on T cells and macrophages suggest a mutual regulation between inflammation and metabolic activity (54, 55). In our study, stimulation of TLR1/2 evidently suppressed OXPHOS activity in chondrocytes. However, the inhibition of OXPHOS and ATP production by rotenone did not induce secretion of the inflammatory cytokines IL-6, IL-8, or G-CSF, suggesting that energy deficiency had little influence on the inflammatory phenotype. This hints towards a unilateral regulation acting from inflammation to OXPHOS. Moreover, rotenone treatment did not affect the expression of *COL2A1*, *ACAN*, and *ADAMTS5*, which indicates that reduction in OXPHOS activity hardly impacted de novo expression of ECM-anabolic or catabolic factors. This is consistent with a study in murine growth-plate chondrocytes reporting that *Sox9*, *Col2*, *Mmp9*, and *Mmp13* mRNA expression was not affected by the reduction of OXPHOS through prolyl hydroxylase 2 deficiency (35). Nevertheless, the potential molecular connection between reduced ATP production and OA pathology remains to be investigated in more detail in future studies.

NO is considered part of the pathophysiology of OA, as high levels of nitrite/ nitrate have been found in the synovial fluid, serum, and urine of OA patients (56–58). TLR1/2 stimulation promoted *NOS2* expression and NO production in chondrocytes, which was consistent with previous studies performed with rodent chondrocytes (16, 59), thus pointing to a potential contribution of TLR signaling in OA development. In turn, NOS inhibition restored impaired OXPHOS activity, rescued ATP production, and dampened the expression of inflammatory and cartilage-degrading factors. Notably, NOS inhibition also prevented the TLR1/2-triggered upregulation of *NFKB* (cf. Fig. 8*A*), a transcription factor that was reported to suppress OXPHOS activity in murine chondrocytes (60). These data further support the notion that NOS inhibitors can be considered potential candidates for disease modulation. Indeed, selective inhibition of iNOS has been shown to delay the progression of OA in humans (61, 62) and of experimentally induced OA in animals (63–65). The effect of iNOS inhibition may well extend beyond reducing NO levels, as it also significantly reduced the levels of prostaglandins at the site of inflammation (66). However, NOS inhibition failed to promote the expression of the ECM-anabolic factors *COL2A1* and *ACAN*, suggesting that NOS inhibition may aid in stopping ongoing cartilage destruction but is less likely to directly promote regeneration.

*Nos2*^−/−^ mice have been used to study the importance of NO in OA pathology and development. However, the observations vary depending on the types of models being used: in a bacterial collagenase Zymosan-induced OA model, cartilage damage was significantly reduced in *Nos2*^−/−^ mice (67), while in a surgical model involving sectioning of the medial collateral ligament and partial medial meniscectomy, *Nos2*^−/−^ mice showed accelerated OA severity (68). We therefore decided on age-associated OA as a naturally occurring, spontaneous OA pathology. While half of the 2-y–old WT mice displayed severe cartilage loss and signs of synovitis, none of the examined *Nos2*^−/−^ mice had such symptoms, indicating that *Nos2* and NO play a significant role in the induction and/or progression of age-related OA. Of note, in the *Nos2*^−/−^ mice studied here the *Nos2* deficiency was not restricted to the chondrocyte lineage. Hence, the reduced age-associated OA in these mice might also, at least in part, result from a potentially decreased production of inflammatory cytokines in synovial cells and not just in chondrocytes. Furthermore, since the activation of some non-TLR pathways can also induce *Nos2* upregulation, TLR-independent effects should not be excluded as potential mechanism of the protection from OA development in the aged *Nos2*^−/−^ mice. Thus, further studies with chondrocyte-specific deletion of *Nos2* and/ or *Tlr2* will help to clarify these points.

Notably, we observed reduced mROS levels and total cellular ROS production, despite an increase in NO production in P3C4-stimulated chondrocyte spheroids. The reduced ROS level most likely resulted from a combination of reduced expression of NOX4 and increased expression of the antioxidant TXNRD1 and the mitochondrial ROS-specific scavenger SOD2 (cf. *SI Appendix*, Fig. S6*C*). A previous study in murine chondrocytes points in the same direction: TLR2/4 deficiency led to enhanced mROS accumulation and reduced SOD2 expression (69), suggesting that TLR2/4 signaling suppresses mROS accumulation by enhancing SOD2 expression. Since NO can be oxidized by O_2_^−^ to form peroxynitrite (ONOO^−^), another ROS component, it is fair to speculate that increased NO production could lead to increased total ROS. However, we observed that both mROS and total cellular ROS production were actually reduced. The reduction of mROS and total cellular ROS were detected by MitoSOX, which is specific for O_2_^−^, and DCFDA, which was thought to be specific for H_2_O_2_ but is actually not specific for any particular ROS according to the recently published guidelines for measuring ROS (70). To reconcile the seeming contradictory observations, we suggest that since the amount of O_2_^−^ (detected by MitoSOX) was at a reduced level in P3C4-stimulated chondrocytes, it is unlikely that it increases the total ROS pool by oxidizing NO to form ONOO^−^, even when there is an increased amount of NO.

Taken together, our study identifies TLR2 signals as potential drivers of OA pathology. Most of the TLR family members and associated signaling adaptor molecules were expressed by human chondrocytes in vivo. This provides the possibility for endogenous TLR agonists, generated by physiological catabolic activities during aging and cartilage injury, to trigger TLR signaling. The inhibition of NOS, which is upregulated not only by TLR2 but also TLR4 and 5 stimulation, largely restored human chondrocyte functionality and protected mice from age-related OA development. Thus, targeting of TLR2 or NOS could be a promising therapeutic strategy to prevent the progression of OA.

## Materials and Methods

### Mice and Assessment of Age-Related OA Severity.

Age-matched male C57BL/6 and *Nos2*^−/−^ mice on C57BL/6 background were maintained under specific-pathogen-free (SPF) conditions until they reached the age of 2 y. Mice were then humanely sacrificed, and hind limb knee joints were removed and processed for OA severity evaluation. For details see *SI Appendix*, *Supplementary Material*. All animal experiments were performed in accordance with the German law for animal protection with permission from the local veterinary offices.

### Patient Samples.

In total, 134 patients (74 women and 60 men; mean age, 70.78 ± 12.01y), who had a clinical and biopsy-proven diagnosis of OA and gave their informed consent for the use of clinical data and samples for research purposes, participated in this study. The study was approved by the Ethics Committee of Charité—Universitätsmedizin Berlin (EA1/032/16) (Table 1).

**Table 1.** Demographic data of cartilage donors

Osteoarthritis patients	No.	Mean age ± SD (y)
Total	134	69.15 ± 11.63
Women	74	67.83 ± 11.22
Men	60	70.78 ± 12.01

### Human Chondrocyte Isolation, Spheroid Culture, and TLR Stimulation.

Femoral condyles of OA patients were collected immediately after removal during knee arthroplasty in the Center for Musculoskeletal Surgery of Charité—Universitätsmedizin Berlin. Cartilage was separated from bone and digested by collagenase II to release chondrocytes. After a round of monolayer culture, chondrocytes were subjected to spheroid generation, hypoxia (4% O_2_) cultivation, and TLR stimulation. For details see *SI Appendix*, *Supplementary Material*.

### Gene Expression Detection.

#### Quantitative reverse transcription PCR.

Samples were blended using a gentleMACS™ device with M tubes (Miltenyi Biotec). mRNA was then isolated using Oligo (dT) magnetic beads (µMACS^TM^ mRNA Isolation Kit, Miltenyi Biotec) following manufacturer’s instructions. cDNA was reverse-transcribed from isolated mRNA using TaqMan reverse transcription reagents (Thermo Fisher Scientific). Expression of target genes was quantified by qPCR using TaqMan™ Fast Advanced Master Mix (Thermo Fisher; 4444556) or Fast SYBR^TM^ Green Master mix reagents and Quant Studio 7 or StepOnePlus™ devices (Thermo Fisher Scientific).

#### RNAScope analysis.

Human cartilage cylinders were collected using a bone extraction SOLIS corer bone device (STRYKER SPINE SAS; 874006). After 24-h fixation with 4% formaldehyde and dehydration with 10%, 20%, and 30% sucrose, cylinders were cryo-embedded with SCEM medium. After sectioning, samples were first subjected to antigen retrieval followed by standard RNAScope procedure according to the RNAScope® Multiplex Fluorescent v2 Assay protocol. Opal570 fluorophore was used for signal visualization by image acquisition using a Zeiss LSM-880 confocal microscope.

#### RNA-sequencing analysis.

Total RNA was isolated using RNeasy Mini Kit (Qiagen; 217004), and cDNA libraries were generated for samples with high RNA integrity (RQN > 8), using the Smart-Seq v4 mRNA Ultra Low Input RNA Kit (Clontech) with up to 10 ng of RNA according to manufacturer’s instructions. Paired-end sequencing (2 × 75 bp) of cDNA libraries was performed on an Illumina NextSeq500 device. Obtained reads were mapped to the hg19 genome (annotation releases: GRCh37.p13) using Tophat2 (71) and Bowtie2 (72) with very sensitive settings. Read counts were determined with featureCounts (73). Further analysis was performed using R (4.0.3).

#### Immunofluorescence.

Human cartilage cylinders were collected and prepared exactly the same as for RNAScope analysis. Sections were permeabilized with 0.3% triton x-100 in PBS for 20 min, and blocked with 10% donkey serum in PBS with 0.05% Tween 20 (PBST) for 30 min at room temperature. TLR2 antibodies (R&D systems) or isotype control antibody were then applied and incubated overnight at 4 °C. Secondary antibody [Donkey anti-Goat IgG (H+L), Life Technologies, A-11058] were used for visualization. Images were acquired using a Zeiss LSM-880 confocal microscope.

#### Flow cytometric analysis.

Chondrocytes were first incubated with IVIG for 15 min to block unspecific binding and stained with LIVE/DEAD™ Fixable Near-IR Dead Cell Stain (Thermo Fisher Scientific) to mark dead cells. Cells were then fixed with 2 % formalin for 10 min and stained with antihuman TLR1 (Abcam; ab59702), TLR2 (Abcam; ab13553), TLR3 (BioLegend; 315010), TLR4 (Enzo Life Science; ALX-804-419F-T100), TLR5 (R&D Systems; FAB6704G), TLR6 (Abcam; ab72362), TLR7 (R&D Systems; IC5875P), TLR8 (R&D Systems; IC8999R), TLR9 (Abcam; ab134369), TLR10 (BioLegend; 354604), and their corresponding isotype controls in 0.05 % Saponin for 30 min. Stained cells were acquired on a FACS Canto II flow cytometer (Becton Dickinson) and analyzed using FlowJo software (version 10.7.1).

### Mito Stress Test Seahorse Assay.

Chondrocyte spheroids were individually placed in the center of Agilent Seahorse XFe96 Spheroid Microplate wells. OCR and ECAR measurements were performed every 5 min prior to and after sequential addition of oligomycin, FCCP, or rotenone/actinomycin A. Data were analyzed using Wave (Agilent).

For more method details see *SI Appendix*, *Supplementary Material*.

### Statistics.

Statistical analysis was performed using GraphPad Prism (v5.02 and v7). Data were first examined for normality. If normal distribution was found, significance was determined using paired or unpaired two-tailed *t* test for two-group comparisons and one-way ANOVA was used for multiple group comparisons. In case of non-normally distributed groups, comparisons were performed using nonparametric tests with corresponding corrections (paired *t* test: Wilcoxon correction; unpaired *t* test: Mann–Whitney *U* test; one-way ANOVA: Friedman test). For comparison of two groups in kinetic analyses, two-way ANOVA was used.

## Supplementary Material

Appendix 01 (PDF)

## Data Availability

The sequence data reported in this paper have been deposited in the Gene Expression Omnibus (GEO) database with an accession no. GSE234821 (74). All other data are included in the article and/or *SI Appendix*.
