# Supplementary material for: NOS inhibition reverses TLR2-induced chondrocyte dysfunction and attenuates age-related osteoarthritis
Source: Proc Natl Acad Sci U S A. 2023 Jul 10;120(29):e2207993120. doi: 10.1073/pnas.2207993120 (PMC10629581; doi:10.1073/pnas.2207993120)
Supplement: Supplementary file 1 — Appendix 01 (PDF) [file pnas.2207993120.sapp.pdf]

Supplementary Information Appendix

NOS inhibition reverses TLR2-induced chondrocyte dysfunction and attenuates age-related osteoarthritis

Ping Shen[a,b,c,1], Sebastian Serve[a,b], Peihua Wu[a,b], Xiaohui Liu[a,b], Yujie Dai[a,b], Nayar Durán-Hernández[a,b], Dan Thi Mai Nguyen[a,b], Michael Fuchs[d], Tazio Maleitzke[e,f,g], Marie-Jacqueline Reisener[e], Maria Dzamukova[a,b], Katrin Nussbaumer[a], Tobias M. Brunner[a,b], Yonghai Li[c], Vivien Holecska[a,b], Gitta A. Heinz[h], Frederik Heinrich[h], Pawel Durek[h], Georgia Katsoula[i,j], Clemens Gwinner[e], Tobias Jung[e], Eleftheria Zeggini[j,k], Tobias Winkler[e,f,l], Mir-Farzin Mashreghi[h], Matthias Pumberger[e], Carsten Perka[e], Max Löhning[a,b,1]

[a]Pitzer Laboratory of Osteoarthritis Research, German Rheumatism Research Center, a Leibniz Institute, 10117 Berlin, Germany

[b]Experimental Immunology and Osteoarthritis Research, Department of Rheumatology and Clinical Immunology, Charité – Universitätsmedizin Berlin, corporate member of Freie Universität Berlin and Humboldt-Universität zu Berlin, 10117 Berlin, Germany

[c]Stem Cell and Biotherapy Engineering Research Center of Henan Province, College of Life Sciences and Technology, Xinxiang Medical University, 453003 Xinxiang, China

[d]Department of Orthopaedic Surgery, University of Ulm, 89081 Ulm, Germany

[e]Center for Musculoskeletal Surgery, Charité – Universitätsmedizin Berlin, corporate member of Freie Universität Berlin and Humboldt-Universität zu Berlin, 10117 Berlin, Germany

[f]Julius Wolff Institute, Berlin Institute of Health at Charité – Universitätsmedizin Berlin, 13353 Berlin, Germany

[g]BIH Charité Clinician Scientist Program, BIH Biomedical Innovation Academy, Berlin Institute of Health at Charité – Universitätsmedizin Berlin, 10178 Berlin, Germany

[h]Systems Rheumatology and Therapeutic Gene Regulation, German Rheumatism Research Center, a Leibniz Institute, 10117 Berlin, Germany

[i]TUM School of Medicine, Technical University of Munich, Graduate School of Experimental Medicine, 81675 Munich, Germany

[j]Institute of Translational Genomics, Helmholtz Zentrum München – German Research Center for Environmental Health, 85764 Neuherberg, Germany

[k]TUM School of Medicine, Technical University of Munich and Klinikum Rechts der Isar, 81675 Munich, Germany

[l]Berlin Institute of Health Center for Regenerative Therapies, Berlin Institute of Health at Charité – Universitätsmedizin Berlin, 13353 Berlin, Germany

Correspondence:

Ping Shen and Max Löhning

Charité – Universitätsmedizin Berlin and Pitzer Laboratory of Osteoarthritis Research, German Rheumatism Research Center (DRFZ), Charitéplatz 1, D-10117 Berlin, Germany
Tel : +49 (0) 30 28 46 07 60
Email: shen@drfz.de; ping.shen@charite.de; max.loehning@charite.de; loehning@drfz.de

Supplementary Material

Methods

Assessment of age-related OA severity in mice

Age-matched male C57BL/6 and *Nos2*$^{-/-}$ mice on C57BL/6 background were maintained under specific-pathogen-free (SPF) conditions until they reached the age of two years. Mice were then humanely sacrificed, and hind limb knee joints were removed and immediately fixed in 4% formaldehyde (Electron Microscopy Sciences; 15713) for two days. After a 9-day decalcification period in decalcification buffer (10% EDTA, pH=7.4), which was exchanged every other day, individual knee joints were embedded in paraffin. For OA severity evaluation, based on the classic OARSI scoring(1), we adapted the modified-OARSI score system from Prof. Tonia Vincent's laboratory at the Kennedy Institute of Rheumatology, Oxford, UK(2). Briefly, the whole knee joint was cut in 4-μm-thick coronal sections from anterior to posterior obtaining 6 to 8 levels separated by approx. 80 μm. One section per level was stained with Safranin-O/Fast green, and all four compartments of the knee articular cartilage (medial and lateral condylar and tibial cartilage) were scored as follows: 0 – no sign of articular cartilage degradation or tissue fibrillation; 1 – cartilage degradation and/or tissue fibrillation; 2 – tissue loss at the surface; 3 – tissue loss up to tidemark; 5 – tissue loss beyond tidemark; 6 – complete loss of tissue. The score of each compartment was summed, obtaining a section score. The three highest section scores were then summed to generate a joint-summed-score. Four individuals performed the scoring independently of each other and in a blinded fashion. The averaged scores of the four

scorers are plotted. For synovitis severity evaluation, the same set of joint sections that were evaluated for OARSI scores were scored, using the modified Krenn score system(3). Four individual scorers performed the scoring independently of each other and in a blinded fashion, and the averaged scores are presented.

Patient samples

In total, 134 patients (74 women and 60 men; mean age, 69.15 ± 11.63 years; please see table below for detailed information), who had a clinical and biopsy-proven diagnosis of OA and gave their informed consent for the use of clinical data and samples for research purposes, participated in this study. Prior to study initiation, ethics approval was obtained from the responsible ethics committee, Ethikkommission der Charité - Universitätsmedizin Berlin (EA1/032/16).

Table 1. Demographic data of cartilage donors

	Osteoarthritis patients	
	Number	Mean age ± SD (years)
Total	134	69.15 ± 11.63
Women	74	67.83 ± 11.22
Men	60	70.78 ± 12.01

Human chondrocyte isolation and spheroid culture

Femoral condyles of OA patients were collected immediately after removal during knee arthroplasty in the Center for Musculoskeletal Surgery of Charité – Universitätsmedizin Berlin. Cartilage was separated from bone and finely diced before incubation at 37°C with 10 mg/ml collagenase II for 4 hours or 1mg/ml collagenase II for 18 hours on a rotator(4). Obtained chondrocytes were subjected to one round of monolayer culture in DMEM/F-12 medium (Gibco supplied with 10% fetal calf serum (Gibco) and 1% penicillin-streptomycin (Gibco). To generate spheroids, adherent chondrocytes were

detached by incubation in accutase (Gibco) and resuspended in DMEM-High Glucose medium (Sigma Aldrich supplemented with 0.1 µM dexamethasone (Sigma Aldrich; D2915), 40 µg/ml L-proline (Sigma Aldrich; P5607), 6.25 µg/ml insulin-transferrin-sodium selenite media supplement (Sigma Aldrich; I1884), 0.1 mg/ml sodium pyruvate (AppliChem; A4859), 1.25 mg/mL bovine serum albumin (Sigma Aldrich; A9418), 1% penicillin-streptomycin (Gibco; 15140-122), 50 µg/ml 2-phospho-L-ascorbic acid trisodium salt (Sigma Aldrich; A8960), 5.35 µg/ml linoleic acid (Sigma Aldrich; L1012), and 10 ng/ml TGF-β1 (Peprotech; 100-21-5). 3-5 x 10^5 chondrocytes were transferred into 15 ml Falcon tubes and centrifuged at 500*g* for 5 min. Spheroids were incubated at 37°C under hypoxic conditions (4% O_2) for indicated durations. Culture medium was exchanged twice per week in a hypoxic chamber workstation (BioSpherix X3, Xvivo system).

TLR stimulation and TLR2 blockade

Chondrocyte spheroids were stimulated with 2 µg/ml of Pam3CSK4, PolyI:C, LPS, Flagellin, Pam2CSK4, Imiquimod, ssRNA, or CpG (InvivoGen), which act as agonists of TLR1/2, 3, 4, 5, 2/6, 7, 8, and 9, respectively. TLR agonists were replenished twice per week. Human 32-mer peptide (FFGVGGEEDITVQTVTWPDMELPLPRNITEGE) was synthesized by the Institute of Medical Immunology, Charité, Berlin. 100 µg/ml 32-mer peptide was added to chondrocyte spheroid cultures. For TLR2 blocking assays, monoclonal anti-human TLR2 antibody (B4H2, 10µg/ml, InvivoGen) was added to chondrocyte spheroids three hours prior to the addition of P3C4.

Chondrocyte spheroid mRNA isolation and quantitative reverse transcription PCR

Control and TLR-stimulated chondrocyte spheroids were transferred in 1 ml Lysis/Binding Buffer (µMACS mRNA Isolation Kit, Miltenyi Biotec) and disrupted using

a gentleMACS™ device with M tubes (Miltenyi Biotec). mRNA was then isolated using Oligo (dT) magnetic beads (μMACS™ mRNA Isolation Kit, Miltenyi Biotec) following manufacturer's instructions. cDNA was reverse-transcribed from isolated mRNA using TaqMan reverse transcription reagents (Thermo Fisher Scientific). Expression of target genes was quantified by qPCR using Fast SYBR™ Green Master mix reagents and Quant Studio 7 or StepOnePlus™ devices (Thermo Fisher Scientific). Normalized expression was determined using the delta-delta Ct method with *ACTB* as a housekeeping gene control. Forward primer (FP) or reverse primer (RP) with the following sequences were obtained from Eurofins Genomics: *ACTB* FP: 5'-CACCCAGCACAATGAAGATCAAGA-3', *ACTB* RP: 5'-CCAGTTTTTAAATCCTGAGTCAAGC-3'; *COL2*A1 FP: 5'-GGAATTCGGTGTGGACATAGG-3', *COL2A1* RP: 5'-ACTTGGGTCCTTTGGGTTTG-3'; *ACAN* FP: 5'-GAATGGGAACCAGCCTATACC-3', *ACAN* RP: 5'-TCTGTACTTTCCTCTGTTGCTG-3'; *MMP3* FP: 5'-TTTTGGCCATCTCTTCCTTCA-3', *MMP3* RP: 5'-TGTGGATGCCTCTTGGGTATC-3'; *ADAMTS5* FP: 5'-GCTCACGAAATCGGACATTTACTT-3', *ADAMTS5* RP: 5'-ACCAAGGTCTCTTCACAGAATTTG-3'; *IL6* FP: 5'-ATGAACTC CTTCTCCACAAGC-3', *IL6* RP: 5'-GTTTTCTGCCAGTGCCTCTTTG-3'; *IL8* FP: 5'-GGCACAAACTTTCAGAGACAGCAG-3', *IL8* RP: 5'-GTTTCTTCCTGGCTCTTGTCCTAG-3'; *G-CSF* FP: 5'-TGAGTGTGCCACCTACAAGC-3', *G-CSF* RP: 5'-GACACCTCCAGGAAGCTCTG-3'; *NOS2* FP: 5'-GTTCTCAAGGCACAGGTCTC-3', *NOS2* RP: 5'-GCAGGTCACTTATGTCACTTATC-3'; *NFKB* FP: 5'- ATGGCTTCTATGAGGCTGAG-3', *NFKB* RP: 5'- GTTGTTGTTGGTCTGGATGC-3'.

Cartilage tissue total RNA isolation and TaqMan qPCR

Femoral condyles of OA patients were collected immediately after removal during knee arthroplasty. Cylinders were collected using a bone extraction SOLIS corer bone device (STRYKER SPINE SAS; 874006). Cartilage was separated from bone and finely diced before snap freezing in liquid nitrogen. Cartilage pieces were further pulverized before being transferred into Trizol buffer and blended using Ultra Turrax (IKA T10 basic). Total RNA was then extracted using the RNeasy Mini Kit (Qiagen; 217004). cDNA was transcribed using TaqMan reverse transcription reagents (Thermo Fisher Scientific; N8080234). TaqMan qPCR were performed in a Quant Studio 7 device utilizing TaqMan™ Fast Advanced Master Mix (Thermo Fisher; 4444556) in combination with the following TaqMan Gene Expression assays: Hs00413978_m1 (*TLR1*), Hs00610101_m1 (*TLR2*), Hs01551078_m1 (*TLR3*), Hs00152939_m1 (*TLR4*), Hs00152825_m1 (*TLR5*), Hs00271977_s1 (*TLR6*), Hs00152971_m1 (*TLR7*), Hs00152972_m1 (*TLR8*), Hs00152973_m1 (*TLR9*), Hs01935337_s1 (*TLR10*), Hs01573837_g1 (*MYD88*), Hs01090712_m1 (*TICAM1*), Hs01019083_m1 (*VDAC1*), Hs03023943_g1 (*ACTB*).

Chondrocyte spheroid total RNA isolation and RNA-sequencing analysis

Chondrocyte spheroids were transferred individually into Trizol buffer and blended using gentleMACS™ (Miltenyi Biotec; 130-096-335). Total RNA was then isolated using RNeasy Mini Kit (Qiagen; 217004). RNA integrity was assessed using a Fragment analyzer (Agilent), and cDNA libraries were generated for samples with high RNA integrity (RQN > 8), using the Smart-Seq v4 mRNA Ultra Low Input RNA Kit (Clontech) with up to 10 ng of RNA according to manufacturer's instructions. Paired-end sequencing (2x75 bp) of cDNA libraries was performed on an Illumina NextSeq500 device. Obtained reads were mapped to the hg19 genome (annotation releases: GRCh37.p13) using Tophat2(5) and Bowtie2(6) with very-sensitive stettings. Read

counts were determined with featureCounts(7). Further analysis was performed using R (4.0.3). Raw data was pre-filtered for genes with ≥ 100 of total read counts across analyzed samples. The DESeq2(8) package was used for normalization, estimation of dispersion, and differential gene expression analysis. A gene was considered as differentially expressed when $|log_2FC| > |log_2(1.3)|$ and P adjusted < 0.05. AnnotationDbi(9) was used for gene annotation and pheatmap(10) and ggplot2(11) for data visualization. Where indicated, batch effect corrections were performed using the limma::removeBatchEffects(12) function with replicates as batches for visualization. Over-representation analysis was performed against biological-process gene sets from the gene ontology resource by using a one-sided version of the Fisher's exact test applying clusterProfiler(13). All expressed genes in the respective conditions were used as a background gene list. The results were simplified in order to reduce overlaps between ontology terms by using the clusterProfiler::simplifyGO function with a cutoff of 0.7. GEO accession: GSE234821.

Bio-plex analysis

Culture supernatants of control or TLR-stimulated spheroids were collected and subjected to Bio-plex assay (Bio-Rad) to quantify the concentration of secreted factors, including IL-6, IL-8, and G-CSF. Bio-plex assay sets of human 27-plex Panel (M50-0KCAF0Y), 3-plex Panel (171-W4001M), 9-plex Panel (171-AM001M), and 4-plex Panel (171-AM002M) from Bio-Rad were used according to manufacturer´s instructions.

Nitric oxide quantification by modified Griess reaction

Culture supernatants of control or TLR-stimulated spheroids were collected and nitrite/nitrate oxidized from NO was measured spectrophotometrically after addition of

Griess reagent (Sigma–Aldrich; G-4410 and InvivoGen) according to manufacturer´s instructions.

Histological analysis

To determine spheroid matrix density, 4-μm-thick sections of formalin-fixed, paraffin-embedded spheroids were first deparaffinised, rehydrated, acidified using 3% acetic acid and subsequently stained with 1% Alcian blue (Sigma-Aldrich; 05500-25G) for 30 min. After dehydration, sections were mounted and images were acquired using Leica Application Suite (LAS Licensing V1.5). To quantify spheroid matrix density, images were analyzed with the software Fiji as described previously(14, 15). Briefly, the basic colour (red, green, blue) of a selected separated 16-bit image was divided and only the blue colour was subjected to intensity quantification. Analyzed parameters included area and area fraction. To visualize the cell nuclei of the spheroid, spheroid sections were additionally stained with Nuclear Fast Red solution (Carl Roth; N069) for 5 min before dehydration.

Human cartilage cylinder preparation, RNAScope analysis, and immunofluoresence

Immediately after obtaining human cartilage resections, cylinders were collected using a bone extraction SOLIS corer bone device (STRYKER SPINE SAS; 874006). After 24-hour fixation with 4% formaldehyde (Electron Microscopy Sciences; 15713) and dehydration with 10%, 20%, and 30% sucrose, cylinders were cryo-embedded with SCEM medium (Section Lab, Japan). The frozen blocks were kept at -80°C until cryosectioning. Kawamoto cryofilms type 3C (16UF) were used for cryosectioning. 10-μm-thick sections were produced. The sections were air-dried in the cryotome for 15 min and stored at -20°C in a slide box with molecular sieves inside. For RNAScope

assays, sections were air-dried at room temperature (RT) for 5 min, washed in PBS for 5 min and baked on a 60°C hot plate for 30 min followed by additional fixation in 4% formaldehyde at 4°C for 15 min. Afterwards, the sections were dehydrated by sequential 5 min incubations in ethanol solutions with gradually decreasing concentrations (50%, 70%, and 100%, repeated once). Sections were air-dried again for 5 min followed by 10 min incubation in hydrogen peroxide and washings in distilled water. The target retrieval buffer was prewarmed (up to 85°C) in a steamer for ~20 min. Slides were submerged into the retrieval buffer and left in the steamer for 10 min. Afterwards, the slides were washed in distilled water, dehydrated in 100% ethanol, and air-dried overnight at RT. Protease III was applied and slides were incubated in a Hybez Oven (ACDBio) for 30 min followed by washings in distilled water. The rest of the RNAScope procedure was performed according to the standard RNAScope® Multiplex Fluorescent v2 Assay protocol using Opal570 fluorophore for signal visualization followed by image acquisition using a Zeiss LSM-880 confocal microscope. For immunofluorescence analysis of TLR2 protein, sections were permeabilized with 0.3% triton x-100 in PBS for 20 min, and blocked with 10% Donkey serum in PBS with 0.05% tween-20 (PBST) for 30 min at room temperature. TLR2 antibodies (R&D systems AF2616) or isotype control antibody were then applied and incubated overnight at 4°C. After washing three times in PBST, sections were incubated with secondary antibody (Donkey anti-Goat IgG (H+L), Life technologies, A-11058) for 1 h at room temperature. After washing three times in PBS, sections were then incubated with DAPI for 10 min at room temperature. Images were acquired using a Zeiss LSM-880 confocal microscope.

Flow-cytometric analysis to detect TLR protein expression

Chondrocytes were isolated from knee femur plateau cartilage tissue by collagenase II digestion overnight. Then they were first incubated with IVIG (Intravenous immunoglobulin, a blood product prepared from the serum of 1.000 to 15.000 donors per batch) for 15 min to block unspecific binding and stained with LIVE/DEAD™ Fixable Near-IR Dead Cell Stain (Thermo Fisher Scientific) to mark dead cells. Cells were then fixed with 2 % formalin for 10 min and stained with anti-human TLR1 (abcam; ab59702), TLR2 (abcam; ab13553), TLR3 (Biolegend; 315010), TLR4 (Enzo Life Science; ALX-804-419F-T100), TLR5 (R&D Systems; FAB6704G), TLR6 (abcam; ab72362), TLR7 (R&D Systems; IC5875P), TLR8 (R&D Systems; IC8999R), TLR9 (abcam; ab134369), TLR10 (Biolegend; 354604), and their corresponding isotype controls in 0.005% Saponin for 30 min. Stained cells were acquired on a FACS Canto II flow cytometer (Becton Dickinson) and analyzed using FlowJo software (version 10.7.1).

GAGs/DNA ratio quantification

Intracellular glycosaminoglycans (GAGs) were measured by using the Glycosaminoglycans Assay Kit according to the manufacturer’s instructions (Glycosaminoglycans Assay kit; Chondrex; no. #6022). DNA was extracted from chondrocyte spheroids by isopropanol precipitation and quantified by NanoDrop Spectrophotometer (Thermo Fisher Scientific; ND-2000C). The ratios of GAGs to DNA were then calculated.

ATP quantification

Spheroid ATP content was quantified by releasing cellular ATP with micro pestles and determining the luminescence after adding luciferase enzyme and luciferin (Abcam). Absorbance at 560nm was measured using a SpectraMax Microplate Reader (Molecular Devices).

MitoSpy, TMRM, MitoSox, and DCFDA staining

Chondrocyte spheroids were dissociated by incubation with 20 mg/ml collagenase II for 15 min at 37°C. A single-cell suspension was obtained by flushing the digested solution through a 26G needle. Cells were then washed and stained for 30 min at 37°C with MitoSpy (Biolegend), TMRM, MitoSOX (Thermo Fisher Scientific), and DCFDA (Abcam; ab113851) diluted in pre-warmed DMEM-High Glucose medium. After washing, LIVE/DEAD™ Fixable Near-IR Dead Cell Stain (Thermo Fisher Scientific) was added to stain dead cells. Samples were acquired on a FACSCanto II (Becton Dickinson) and data were analyzed with FlowJo software (version 10.7.1).

Mito Stress Test seahorse assay

After 4-day stimulation, control- and TLR-stimulated chondrocyte spheroids were individually placed in the centre of Agilent Seahorse XFe96 Spheroid Microplate wells, which were precoated with poly-D-lysine and contained prewarmed assay buffer. Microplates were kept at 37°C in a non-CO_2 until loading of spheroids. OCR and ECAR measurements were performed every 5 min prior to and after sequential addition of oligomycin, FCCP or Rotenone/Antimycin A. Data were analyzed using Wave (Agilent).

Additional reagents

N-Nitro-L-arginine methyl ester hydrochloride (L-NAME) used in this study was purchased from Abcam (ab120136) and Sigma-Aldrich (N5751-1G). Rotenone was purchased from Cayman Chemical (Cay13995-1).

Statistics

Statistical analysis was performed using GraphPad Prism (v5.02 and v7). Data were first examined for normality. If normal distribution was found, significance was determined using paired or unpaired two-tailed *t* test for two-group comparisons and one-way ANOVA was used for multiple group comparisons. In case of non-normally

distributed groups, comparisons were performed using nonparametric tests with corresponding corrections (paired *t* test: Wilcoxon correction; unpaired *t* test: Mann Whitney U test; One-way ANOVA: Friedman test). For comparison of two groups in kinetic analyses, Two-way ANOVA was used.

Study approval

The study was approved by the Ethics Committee of Charité – Universitätsmedizin Berlin (EA1/032/16).

Author contributions

PS and ML designed the research. PS, PW, XL, YD, NDH, DTMN, MD, KN, YL, and VH performed the experiments and analyzed the data. MFM, SS, TMB, GAH, FH, PD, GK, and EZ conducted the RNA-sequencing and the corresponding computational data analysis. MF, TM, MJR, CG, TJ, TW, MP, and CP coordinated patient sample collection. PS and ML wrote the paper.

Acknowledgments

This work was supported by the Willy Robert Pitzer Foundation (Pitzer Laboratory of Osteoarthritis Research), the Dr. Rolf M. Schwiete Foundation (Osteoarthritis Research Program), the Einstein Center for Regenerative Therapies (EZ-2016-289), the German Research Foundation (DFG; grants LO 1542/4-1 and LO 1542/5-1), the German Federal Ministry of Education and Research (BMBF; grant 01KC2011C) the National Natural Science Foundation of China (81671619), the European Regional Development Fund (ERDF 2014–2020, EFRE 1.8/11), and the state of Berlin. We thank the Microscopy Core Facility of Max Planck Institute for Infection Biology and Dr. Volker Brinkmann for their help on histological analysis. XL and YD were supported by scholarships from the China Scholarship Council. We thank Adrian Madrigal, Lisa Grunwald, Carola Rüster, Katrin Lehmann, and Isabel Panse for experimental

assistance; Caroline Peine, Dominik Niesen, Anna Rapp, and Valerie Plajer for scientific discussions; Philippe Saikali, Stefan H.E. Kaufmann, and Arturo Zychlinsky for provision of materials; Sven Geißler and Georg N. Duda for advice on the chondrocyte spheroid culture system; Ahmed N. Hegazy for providing chemicals; and Jan Phillip Weber for graphic processing.

References

1. S. S. Glasson, M. G. Chambers, W. B. Van Den Berg, C. B. Little, The OARSI histopathology initiative - recommendations for histological assessments of osteoarthritis in the mouse. *Osteoarthritis Cartilage* 18 Suppl 3, S17-23 (2010).
2. X. Tang *et al.*, Connective tissue growth factor contributes to joint homeostasis and osteoarthritis severity by controlling the matrix sequestration and activation of latent TGFbeta. *Ann Rheum Dis* **77**, 1372-1380 (2018).
3. J. S. Lewis *et al.*, Acute joint pathology and synovial inflammation is associated with increased intra-articular fracture severity in the mouse knee. *Osteoarthritis Cartilage* **19**, 864-873 (2011).
4. Shen P, Wu P, Maleitzke T, Reisener M, Heinz G, Heinrich F, Durek P, Gwinner C, Winkler T, Pumberger M, Perka C, Mashreghi MF, Löhning M. Optimization of chondrocyte isolation from human articular cartilage to preserve the chondrocyte transcriptome. *Front. Bioeng. Biotechnol. 10, 1046127 (2022).*
5. D. Kim *et al.*, TopHat2: accurate alignment of transcriptomes in the presence of insertions, deletions and gene fusions. *Genome Biol* **14**, R36 (2013).
6. B. Langmead, S. L. Salzberg, Fast gapped-read alignment with Bowtie 2. *Nat Methods* **9**, 357-359 (2012).
7. Y. Liao, G. K. Smyth, W. Shi, featureCounts: an efficient general purpose program for assigning sequence reads to genomic features. *Bioinformatics* **30**, 923-930 (2014).
8. M. I. Love, W. Huber, S. Anders, Moderated estimation of fold change and dispersion for RNA-seq data with DESeq2. *Genome Biol* **15**, 550 (2014).
9. H. Pagès, M. Carlson, S. Falcon, N. Li (2020) AnnotationDbi: Manipulation of SQLite-Based Annotations in Bioconductor. (Available online:).
10. R. Kolde (2018) pheatmap: Pretty Heatmaps. (https://cran.mtu.edu/web/packages/pheatmap/index.html).
11. Hadley W. *et al.* (2021) ggplot2: Create Elegant Data Visualisations Using the Grammar of Graphics. (https://ggplot2.tidyverse.org, https://github.com/tidyverse/ggplot2).
12. M. E. Ritchie *et al.*, limma powers differential expression analyses for RNA-sequencing and microarray studies. *Nucleic Acids Res* **43**, e47 (2015).
13. G. Yu, L. G. Wang, Y. Han, Q. Y. He, clusterProfiler: an R package for comparing biological themes among gene clusters. *OMICS* **16**, 284-287 (2012).
14. J. Schindelin *et al.*, Fiji: an open-source platform for biological-image analysis. *Nat Methods* **9**, 676-682 (2012). Accessed date: 05.14.2021.
15. B. Eggerschwiler, D. D. Canepa, H. C. Pape, E. A. Casanova, P. Cinelli, Automated digital image quantification of histological staining for the analysis of the trilineage

differentiation potential of mesenchymal stem cells. *Stem Cell Res Ther* **10**, 69 (2019). Accessed date: 05.14.2021.

Supplementary Figures

Figs. S1-10

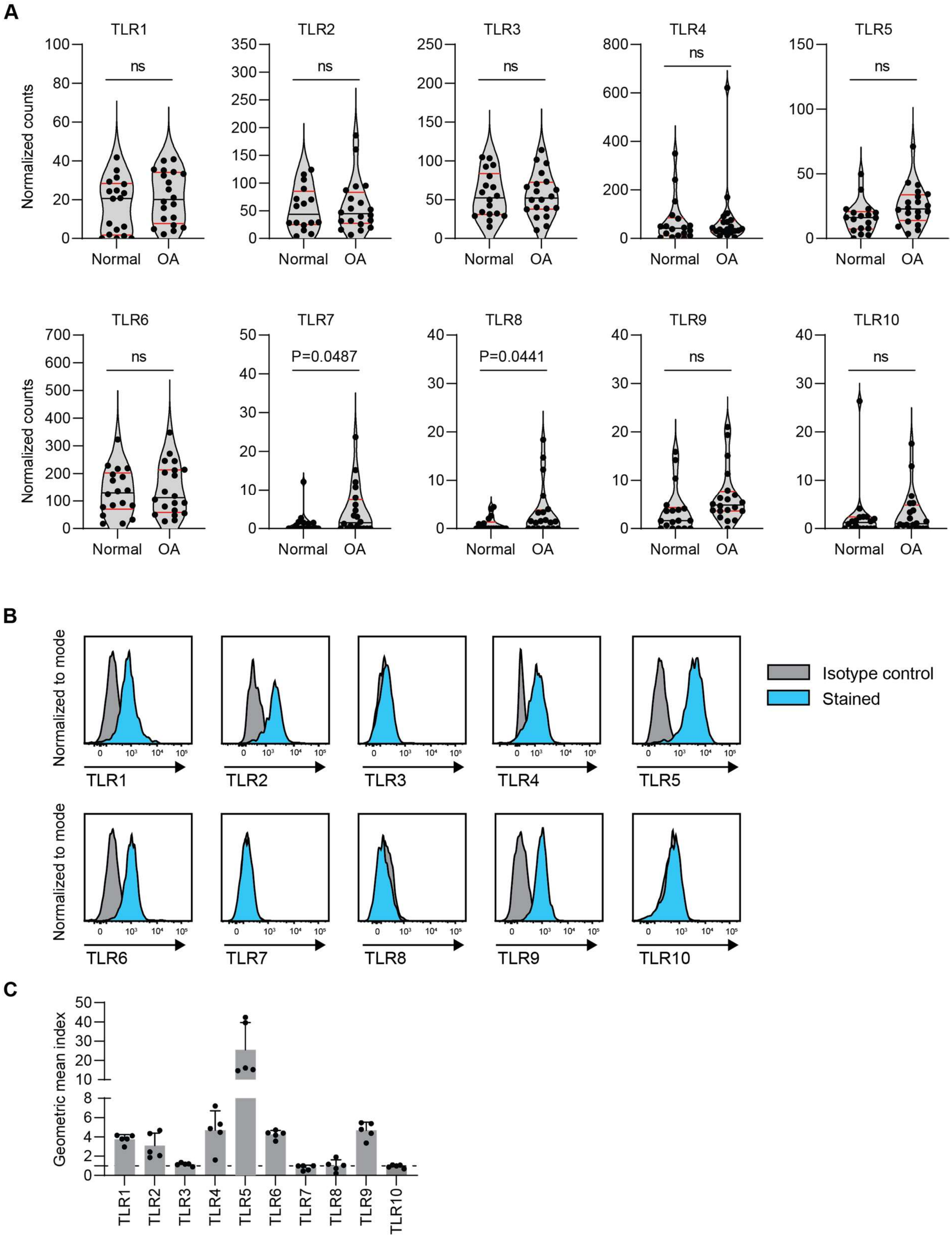

Fig. S1. Human chondrocytes express various TLRs at both mRNA and protein level. (A) The RNA-seq data set GSE114007 from human knee cartilage was analyzed for TLR gene expression(24). Briefly, normal human knee cartilage tissues were collected from individuals without history of joint disease or trauma and processed within 24–48 hours post mortem. OA-affected cartilage was harvested from the tissue removed during knee replacement surgery. Cartilage was stored at −20ºC immediately after harvest until RNA extraction. RNA-seq analysis was conducted, and normalized counts of TLR1 to TLR10 were plotted (Normal: n=18; OA: n=20; mean ± SD). Data were analyzed with unpaired two-tailed *t* test. P-values > 0.05 are considered as non-significant (ns). (B and C) Chondrocytes were isolated from cartilage tissue of human knee femur plateaus by collagenase II digestion overnight. Then cells were fixed and stained with antibodies to TLR1, 2, 3, 4, 5, 6, 7, 8, 9, and 10 intracellularly, in parallel with their corresponding isotype control, and analyzed by FACS. (B) Representative staining histograms for each TLR, together with its corresponding isotype control. (C) Geometric mean index (geometric mean fluorescence intensity (MFI) of TLR-stained cells divided by the geometric MFI of the respective isotype-stained cells) from five individual donors are plotted (n=5, mean + SD).

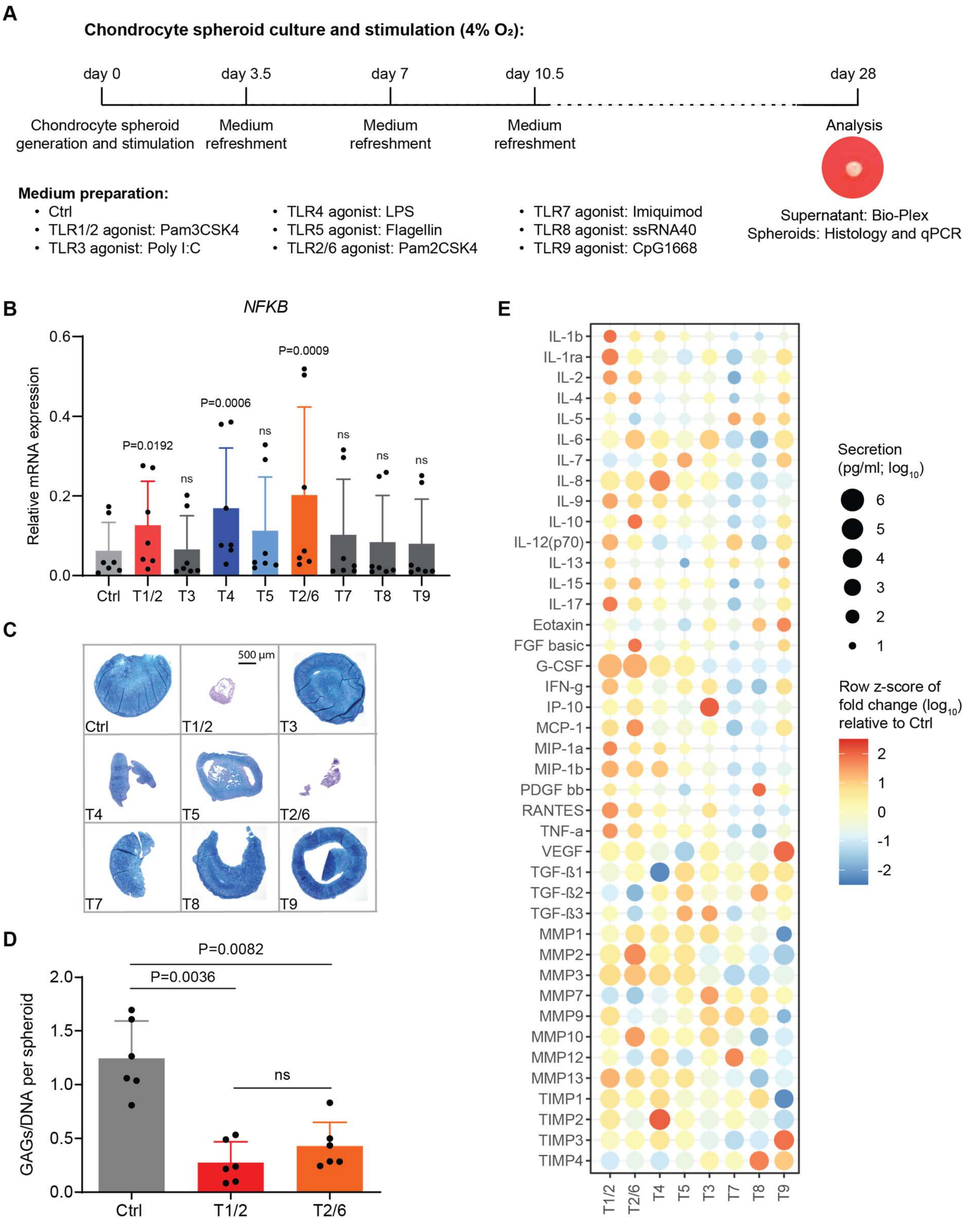

Fig. S2. Human chondrocytes respond differently to distinct TLR stimulation. (A) A schematic graph illustrating the chondrocyte spheroid culture, stimulation, and analysis timeline. (B) Human chondrocyte spheroids were stimulated with agonists of TLR1/2 to TLR9 for 18 hours before being lysed for mRNA isolation. The expression of *NFKB*

was assessed by qPCR (n=7, mean + SD). Data were analyzed using Friedman test to compare each TLR-stimulated sample to Ctrl sample. P-values > 0.05 are considered as non-significant (ns). (C) Chondrocyte spheroids were stimulated with agonists of TLR1/2 to TLR9 for 28 days before being fixed, paraffin-embedded, sectioned, and stained with Alcian blue and Nuclear Fast red. Representative images of each condition from one patient are shown. (D) Chondrocyte spheroids were stimulated with agonists of TLR1/2 or TLR2/6 for 28 days. GAGs and DNA content in each spheroid were measured. Plotted data present the ratios of GAGs to DNA per spheroid (n=6, mean + SD). Data were analyzed using One-Way ANOVA. (E) Supernatants were harvested on day 3.5 and analyzed by Bio-Plex to determine the concentrations of inflammatory cytokines, chondrogenic cytokines (e.g., transforming growth factor beta (TGFβ)), catabolic factors (e.g., MMPs), and catabolism inhibitory factors (e.g., TIMPs) (n=4, mean).

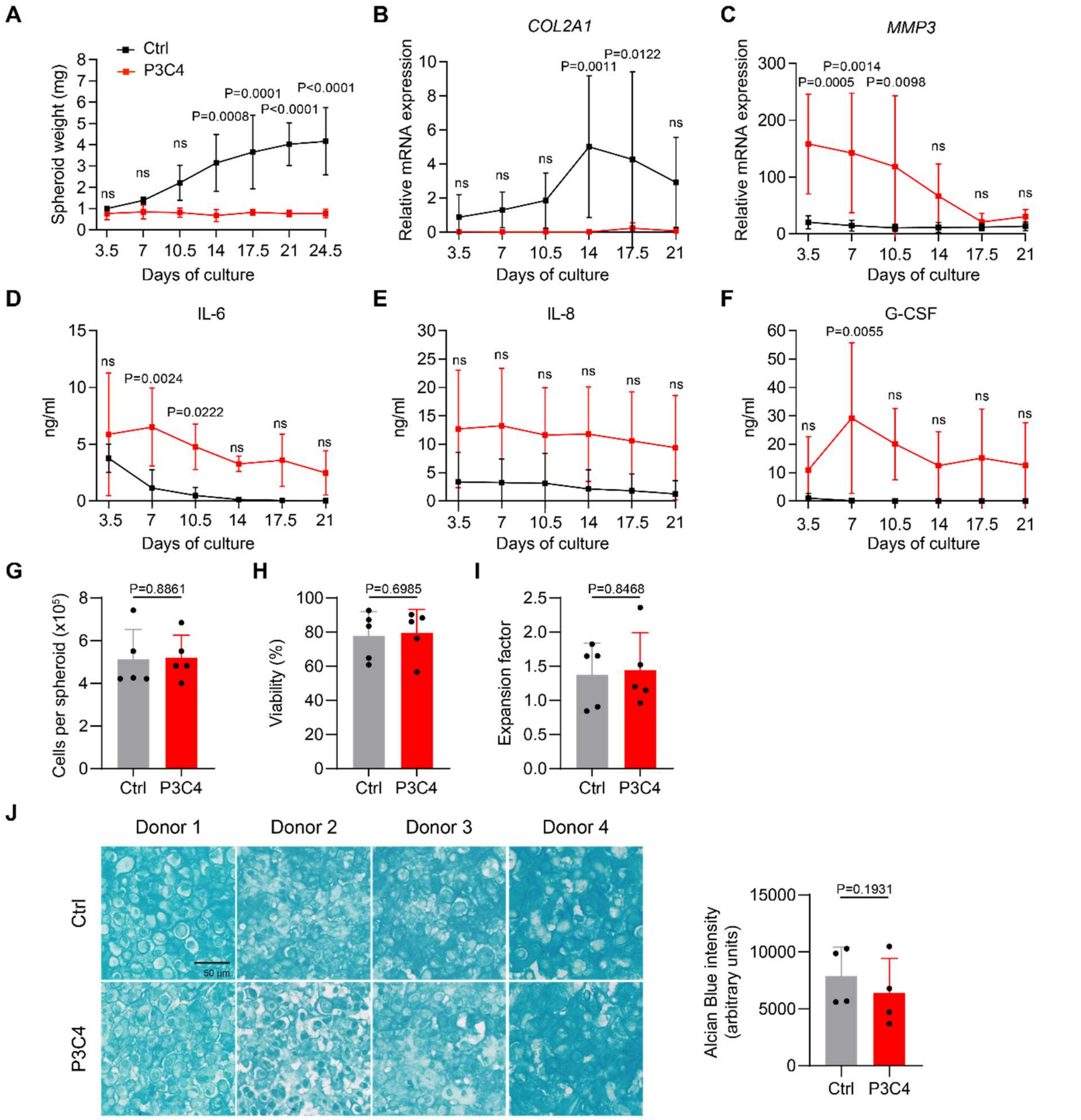

Fig. S3. TLR1/2 stimulation affects chondrocyte spheroids during both long-term (24.5 days) and short-term (3.5 days) stimulation. (A-F) Chondrocyte spheroids were generated and divided into two groups: unstimulated (Ctrl) and P3C4-stimulated (P3C4). Over a 24.5-day culture period, every 3.5 days, one pair of Ctrl and P3C4 samples were taken to determine spheroid weight (A, n=4); *COL2A1* and *MMP3* mRNA expression (B and C, n=6); and IL-6, IL-8, and G-CSF secretion (D and F, n=5). Data (mean ± SD) were analyzed using two-way ANOVA to compare the difference between Ctrl and P3C4-stimulated samples at each timepoint. P-values > 0.05 are

considered as non-significant (ns). (G-I) Chondrocyte spheroids were generated and cultured with or without P3C4 for 3.5 days. Individual spheroids were then dissociated to release single chondrocytes. Cell number per spheroid (G), cell viability (H), and cell expansion factor (I) were assessed. Data (n=5, mean + SD) were analyzed using paired two-tailed *t* test. (J) Chondrocyte spheroids were generated and cultured with or without P3C4 for 3.5 days before being processed for Alcian blue staining. Representative images (left) and relative quantifications of Alcian blue intensity (right) are shown. Data (n=4, mean + SD) were analyzed using paired two-tailed *t* test.

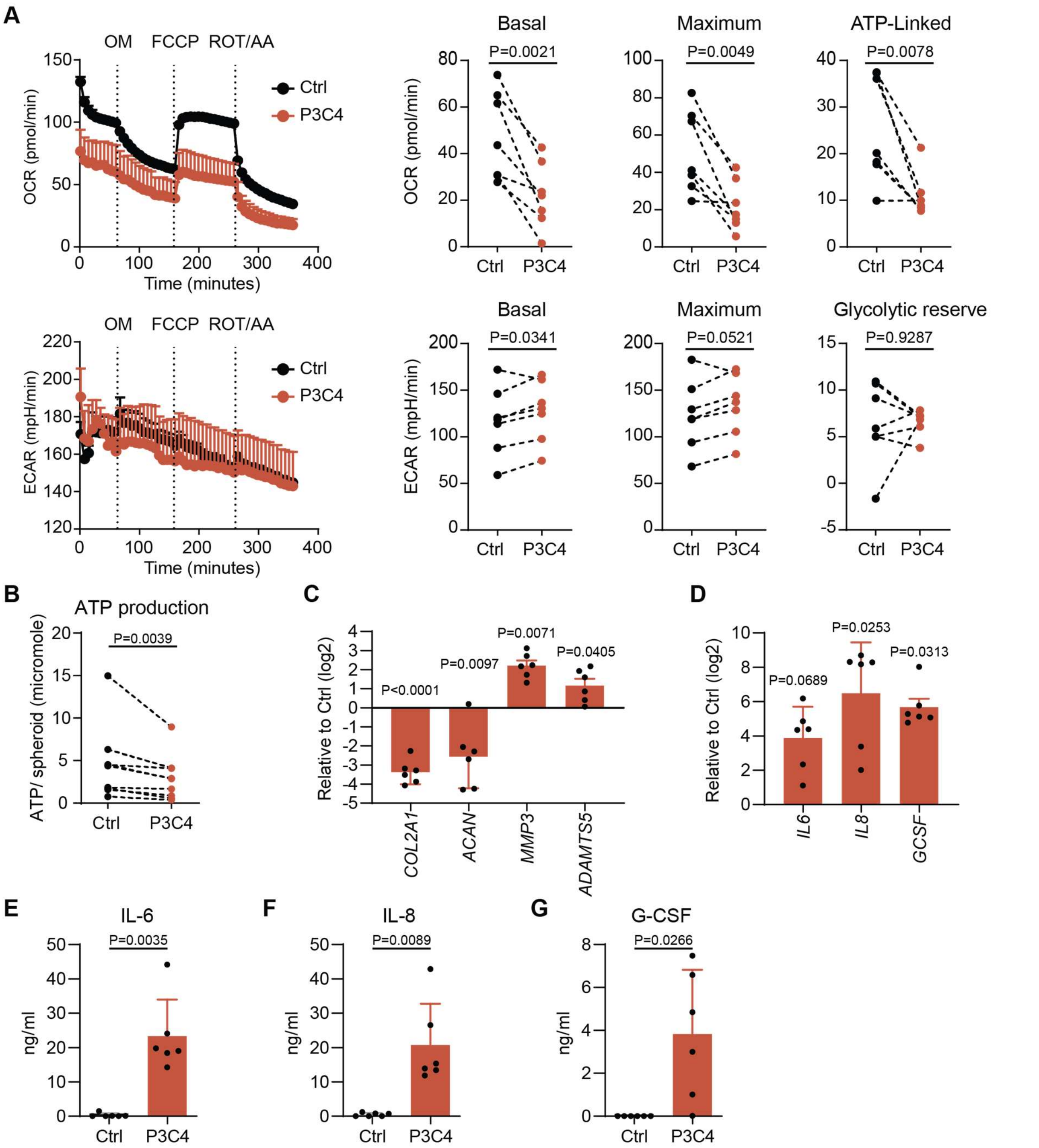

Fig. S4. TLR1/2 stimulation impairs mitochondrial respiration and induces inflammation in mature chondrocyte spheroids similarly as in freshly established spheroids. Human chondrocyte spheroids were cultured for 28 days without stimuli to establish maturity before adding P3C4 and keeping the stimulation for 3.5 days. (A) Spheroids were transferred individually into a Seahorse Spheroid Microplate and analyzed for their OXPHOS (upper panels) and glycolytic (lower panels) activity using Mito Stress Test kits (n=7). Data were analyzed using paired two-tailed *t* test. (B) Spheroids were

grinded and lysed individually to release ATP, which were immediately quantified and calculated as concentration of ATP per spheroid (n=9). (C and D) Spheroids were lysed for mRNA isolation and the expression of *COL2A1*, *ACAN*, *MMP3*, *ADAMTS5*, *IL6*, *IL8*, and *GCSF* was analyzed by qPCR and normalized to Ctrl (n=6, mean ± SD). (E-G) Supernatants were collected and the concentrations of IL-6, IL-8, and G-CSF were determined by Bio-Plex (n=6, mean + SD). B-G, Data were analyzed using two-tailed Wilcoxon matched-pairs test.

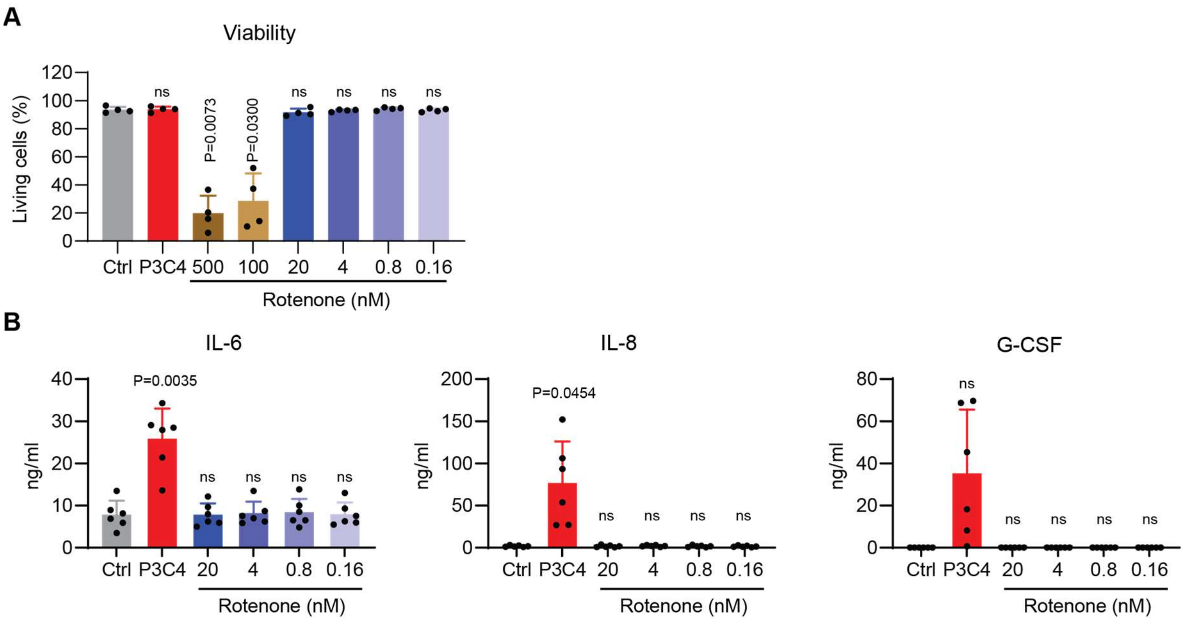

Fig. S5. Inhibition of mitochondrial respiration has no effect on inflammatory cytokine production. Chondrocyte spheroids were generated and stimulated with P3C4 for 3.5 days. Decreasing concentrations of rotenone were added to inhibit mitochondrial respiration. (A) Single chondrocytes were recovered from spheroids to evaluate cell viability. Data (n=4, mean + SD) were analyzed using one-way ANOVA followed by Dunnett´s post-test. (B) The concentrations of IL-6, IL-8, G-CSF were determined in the supernatants. Data (n=6, mean + SD) were analyzed using one-way ANOVA followed by Dunnett´s post-test. P-values > 0.05 are considered as non-significant (ns).

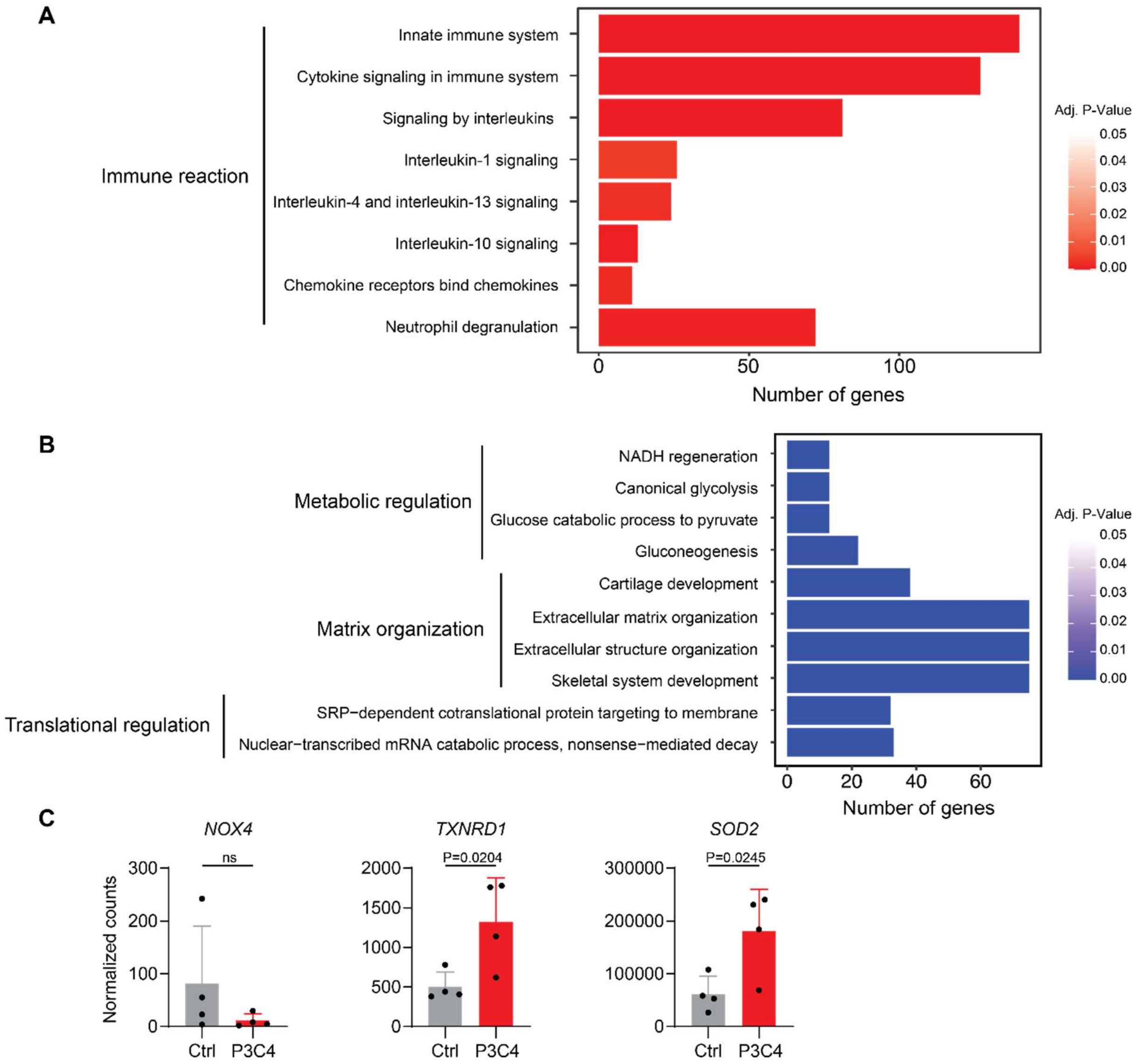

Fig. S6. TLR1/2 induces upregulation of gene clusters in cytokine signaling and downregulation in cartilage matrix organization, metabolic activity, and translational regulation. RNA-seq analysis was performed on human chondrocyte spheroids that were cultured with or without P3C4 for 3.5 days. Overrepresentation analysis of genes upregulated and downregulated by P3C4 stimulation was performed employing "gene ontology biological processes" gene sets. Selected results with adjusted P-values <0.05 are plotted. Barplots of selected gene sets in which upregulated (A) and downregulated (B) genes are overrepresented. Bar length represents the size of the overlap between the differentially up- or downregulated genes and the respective gene set and the color representing the adjusted P-value. (C) Normalized reads of *NOX4*,

TXNRD1, and *SOD2* are plotted (n=4, mean + SD). Data were analyzed using paired two-tailed *t* test. P-values > 0.05 are considered as non-significant (ns).

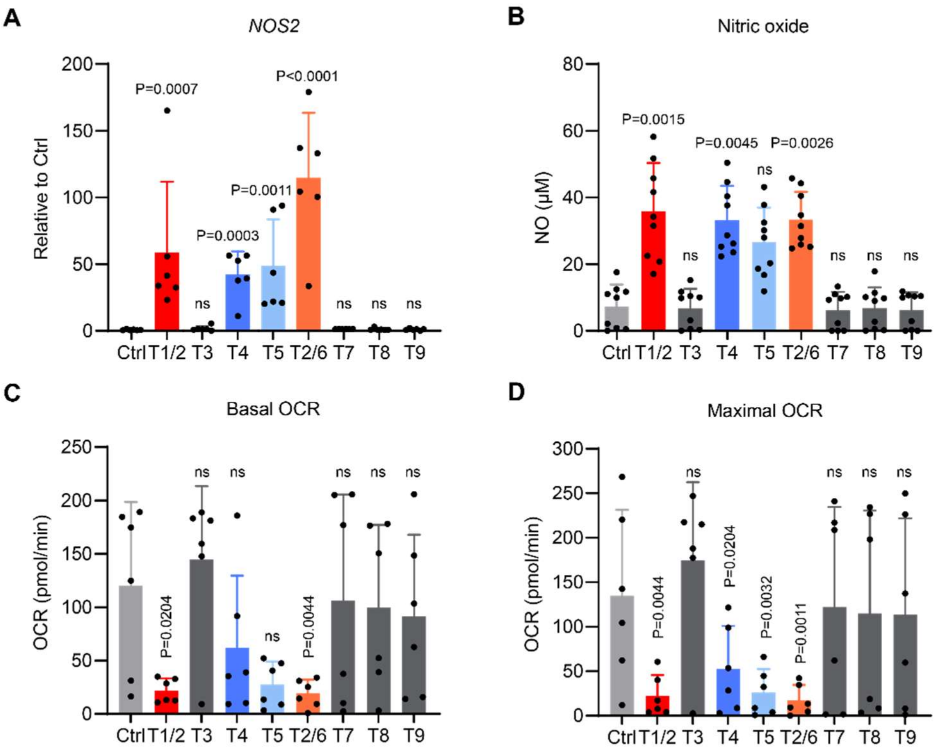

Fig. S7. *NOS2* expression and NO production are inversely correlated with OXPHOS activity upon distinct TLR stimulation. Chondrocyte spheroids were stimulated with agonists of TLR1/2, 3, 4, 5, 6, 7, 8, and 9 individually for 3.5 days. (A) Spheroids were lysed for mRNA isolation. The expression of *NOS2* was assessed by qPCR (n=6, mean + SD). (B) Supernatants of the culture were collected and the concentration of nitric oxide was quantified by Griess reactions (n=9, mean + SD). (C and D) Spheroids were analyzed for their OXPHOS activity using Mito Stress Test kits (n=6, mean + SD). Data were analyzed using Friedman test to compare each TLR-stimulated condition to the respective Ctrl. P-values > 0.05 are considered as non-significant (ns).

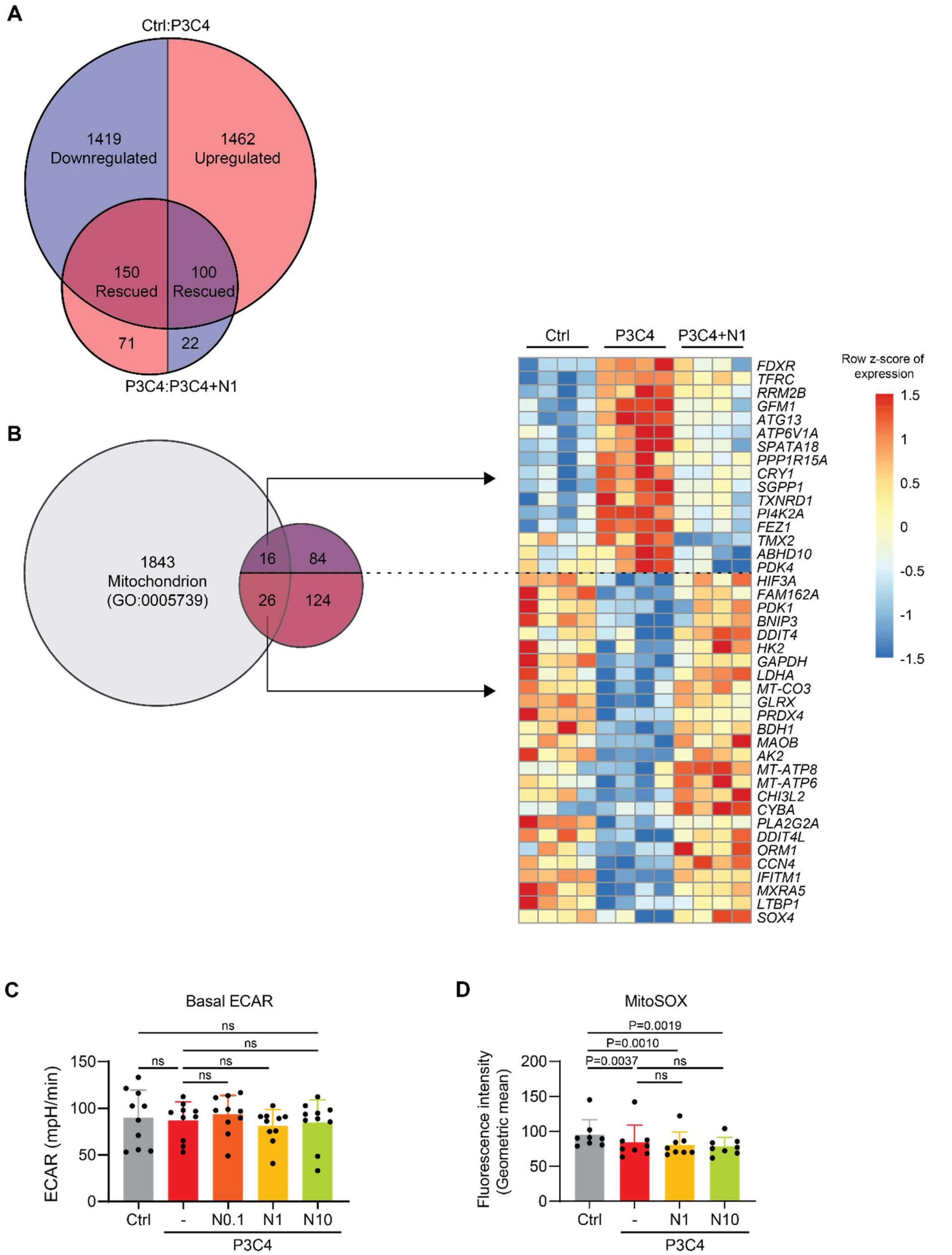

Fig. S8. NOS inhibition rescues the TLR1/2-affected expression of numerous mitochondrial function-related genes, without affecting glycolysis or ROS accumulation.

RNA-seq analysis was performed for spheroids that were unstimulated (Ctrl) or stimulated with either P3C4 or P3C4+N1. (A) Venn diagram depicting the number of genes that are either downregulated by P3C4 stimulation (1419) and upregulated (rescued) by additional treatment with L-NAME (150) or upregulated by P3C4 stimulation (1462) and downregulated (rescued) by additional treatment with L-NAME (100). Differential expression is defined as |log2 Fold Change| > 1.3 and an adjusted P-value < 0.05. (B) Row-clustered heatmap of the rescued genes that also overlap with the gene ontology gene set of mitochondrion genes (GO:0005739). Chondrocyte spheroids were cultured with or without P3C4. In parallel, increasing amounts of the NOS inhibitor L-NAME were added to the P3C4-stimulated cultures (N0.1: 0.1 mM; N1: 1 mM; N10: 10 mM). (C) On day 3.5, spheroids were analyzed for their basal ECAR and glycolysis efficiency using Mito Stress Test kits (n=10, mean + SD). (D) Single chondrocytes were recovered from spheroids using collagenase II to determine mROS accumulation as indicated by the intensity of MitoSOX staining. Data show geometric mean fluorescence intensity of MitoSOX (n=8, mean + SD). (C and D) Data were analyzed using Friedman test. P-values > 0.05 are considered as non-significant (ns).

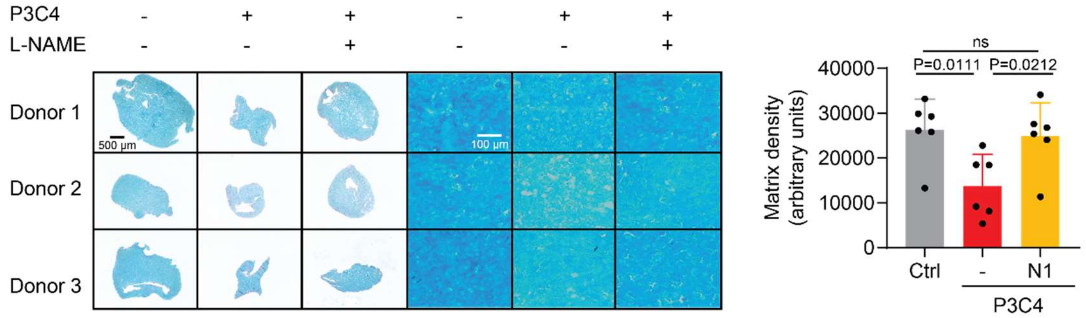

Fig. S9. NOS inhibition restores spheroid matrix density. Ctrl, P3C4-, and P3C4+N1-treated spheroids were cultured for 28 days. At the end of the culture, extracellular matrix density was assessed by Alcian blue staining. Global and zoom-in views of the spheroid sizes and matrix densities (left) and quantification of the matrix densities (right) are shown. Data (n=6, mean + SD) were analyzed using one-way ANOVA followed by Tukey´s post-test. P-values > 0.05 are considered as non-significant (ns).

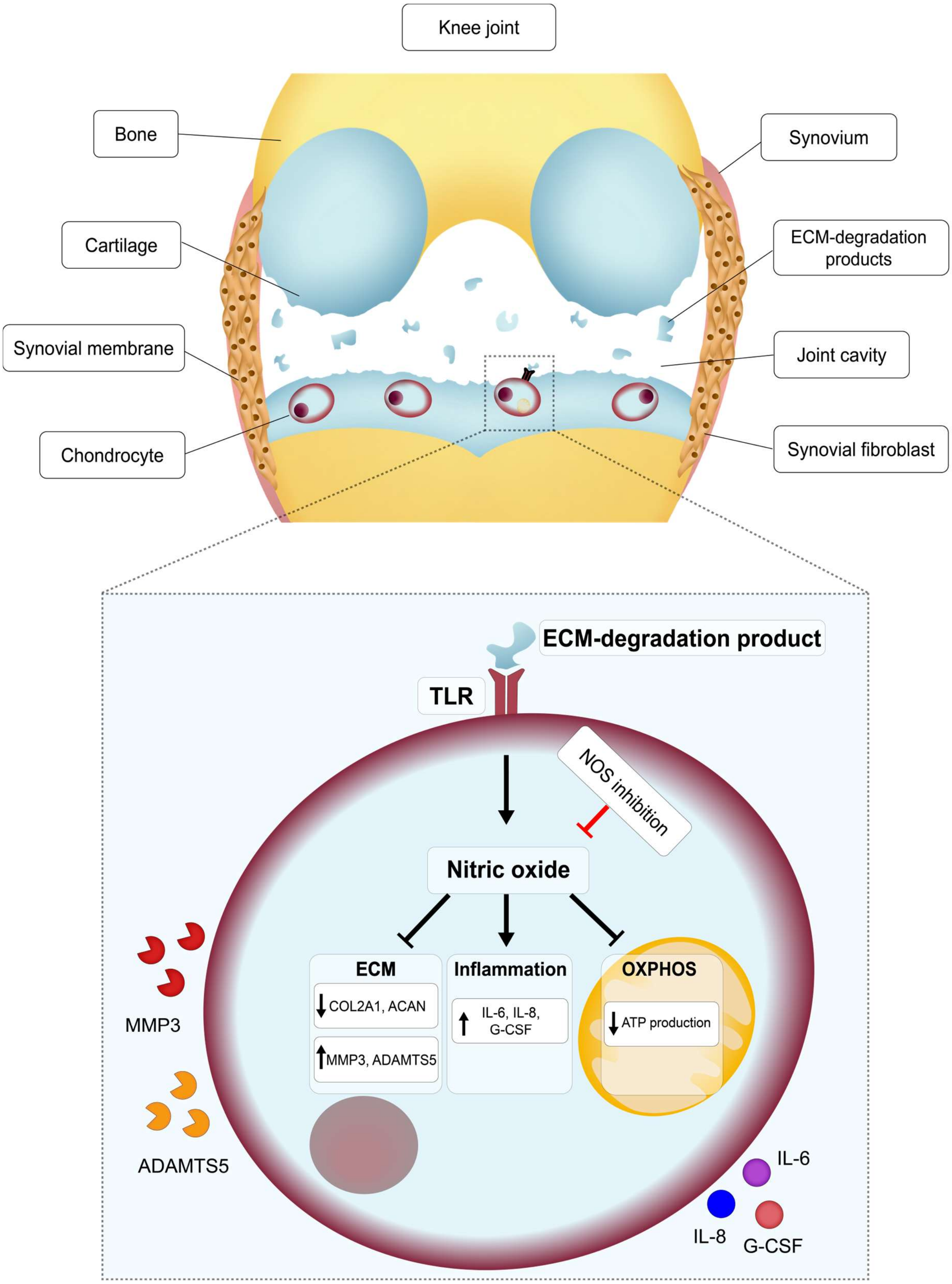

Fig. S10/ Graphical abstract

Cartilage matrix deterioration, a hallmark of osteoarthritis (OA), can initiate an intracapsular accumulation of small degradation products. Some of these degradation

products can function as damage-associated molecular patterns to activate cells through Toll-like receptors (TLR). Chondrocytes residing in cartilage are exposed to these endogenous TLR agonists, and thus can be activated and increase their secretion of matrix-digestive enzymes like MMP3, ADAMTS5, and inflammatory cytokines such as IL-6, to further worsen cartilage integrity. In this study, we show that OA chondrocytes indeed express several of the TLR family members *in vivo*, in particular TLR2. Stimulation of TLR1/2 and TLR2/6 suppressed the growth of chondrocyte spheroids most drastically. This growth suppression was associated with decreased anabolism and increased catabolism of extracellular matrix (ECM), an enhanced inflammatory status with increased production of nitric oxide (NO), and impaired mitochondrial function. Inhibition of nitric oxide synthase (NOS) rescued the chondrocytes' energy deficit, suppressed the inflammation, and readjusted the anabolic-catabolic balance of ECM. Finally, NOS inhibition partially rescued chondrocyte spheroid growth and protected mice from age-related OA development.
